# Evaluation of science advice during the COVID-19 pandemic in Sweden

**DOI:** 10.1057/s41599-022-01097-5

**Published:** 2022-03-22

**Authors:** Nele Brusselaers, David Steadson, Kelly Bjorklund, Sofia Breland, Jens Stilhoff Sörensen, Andrew Ewing, Sigurd Bergmann, Gunnar Steineck

**Affiliations:** 1 Centre for Translational Microbiome Research, Karolinska Institutet, Stockholm, Sweden; 2 Global Health Institute, Antwerp University, Antwerp, Belgium; 3 Department of Head and Skin, Ghent University, Ghent, Belgium; 4 Science Forum COVID-19, Umeå, Sweden; 5 VanaTech Behavioural Science, Älvkarleby, Sweden; 6 Freelance Journalist, Stockholm, Sweden; 7 Freelance Journalist, Washington, DC USA; 8 Oskarström Primary Care, Halmstad, Sweden; 9 School of Global Studies, Gothenburg University, Gothenburg, Sweden; 10 Department of Chemistry and Molecular Biology, Gothenburg University, Gothenburg, Sweden; 11 Department of Philosophy and Religious Studies, Norwegian University of Science and Technology, Trondheim, Norway; 12 Clinical Cancer Epidemiology, Department of Oncology, Gothenburg University, Gothenburg, Sweden

**Keywords:** Health humanities, Science, technology and society, Social policy

## Abstract

Sweden was well equipped to prevent the pandemic of COVID-19 from becoming serious. Over 280 years of collaboration between political bodies, authorities, and the scientific community had yielded many successes in preventive medicine. Sweden’s population is literate and has a high level of trust in authorities and those in power. During 2020, however, Sweden had ten times higher COVID-19 death rates compared with neighbouring Norway. In this report, we try to understand why, using a narrative approach to evaluate the Swedish COVID-19 policy and the role of scientific evidence and integrity. We argue that that scientific methodology was not followed by the major figures in the acting authorities—or the responsible politicians—with alternative narratives being considered as valid, resulting in arbitrary policy decisions. In 2014, the Public Health Agency, after 5 years of rearrangement, merged with the Institute for Infectious Disease Control, with six professors leaving between 2010 and 2012 going to the Karolinska Institute. With this setup, the authority lost scientific expertise. The Swedish pandemic strategy seemed targeted towards “natural” herd-immunity and avoiding a societal shutdown. The Public Health Agency labelled advice from national scientists and international authorities as extreme positions, resulting in media and political bodies to accept their own policy instead. The Swedish people were kept in ignorance of basic facts such as the airborne SARS-CoV-2 transmission, that asymptomatic individuals can be contagious and that face masks protect both the carrier and others. Mandatory legislation was seldom used; recommendations relying upon personal responsibility and without any sanctions were the norm. Many elderly people were administered morphine instead of oxygen despite available supplies, effectively ending their lives. If Sweden wants to do better in future pandemics, the scientific method must be re-established, not least within the Public Health Agency. It would likely make a large difference if a separate, independent Institute for Infectious Disease Control is recreated. We recommend Sweden begins a self-critical process about its political culture and the lack of accountability of decision-makers to avoid future failures, as occurred with the COVID-19 pandemic.

## Introduction

The launch of The Royal Swedish Academy of Sciences in 1739 can be seen as the starting point for >280 years of cooperation between the political power, civil servants, and the scientific community in Sweden (Andréasson et al., 1997, 2021i). Since 1749, Sweden has a population-based cause-of-death registration mandated by law, providing reliable statistics on which preventive measures can be based (Andréasson et al., 1997, 2018). Sweden has thus placed itself at the forefront internationally when it comes to preventing disease and death (Andréasson et al., 1997), and seemed well equipped and prepared for the COVID-19 pandemic.

To look at a few success stories (Andréasson et al., 1997):

- During the 1800s, the Swedish state took initiatives to replace women who had traditionally assisted in childbirth in rural areas by scientifically trained midwives, leading to a clear reduction in maternal mortality.
- During the 1950s, free evidence-based maternal healthcare was launched for all women in Sweden, resulting in among the lowest perinatal mortality numbers globally for several decades
- During the 1960s, actions were taken towards strict legislation that would protect children from accidents at home, in traffic, and at construction sites. This legislation was based on domestic scientific studies.
- One of the latest examples of a successful collaboration between the scientific community and government has been the response to the HIV pandemic. A delegation of the country’s foremost experts was formed, which was in a continuous dialogue with the government. The work to prevent the spread of infection and lessen the stigmatisation of those infected with HIV was successful in an international perspective.

When the COVID-19 pandemic hit Europe, this sparsely populated Nordic country stood out from the beginning (Murray, 2020; Orlowski and Goldsmith, 2020; Habib, 2020; Esaiasson et al., 2021; Lindström, 2020b), with an apparent unhurried and less restrictive strategy compared to the rest of the continent (Hale et al., 2021). Several sources highlighted “Swedish exceptionalism” as the underlying reason for this diversion, going against international and scientific advice (Nygren and Olofsson, 2021; Granberg et al., 2021). While other European nations went into strict lockdown mid-March, Sweden banned public gatherings of >500 people, and >50 at the end of March (Orlowski and Goldsmith, 2020). Officially, the Swedish strategy was centred around individual responsibility (Nygren and Olofsson, 2020), and not overwhelming healthcare systems. The aim was to protect risk groups (including elderly) and to limit the consequences for the individual and society—a so-called mitigation strategy (Walker et al., 2020; Lindström, 2020b, Sayers, 2020). During 2020, most schools were never pro-actively closed to hinder virus spreading (with compulsory physical attendance required by law in Sweden and no option for distance or home learning) (Orlowski and Goldsmith, 2020). Distance learning was however introduced for older teenagers and university students (Orlowski and Goldsmith, 2020). The Swedish strategy has been principally based on recommendations and voluntary measures, for example working from home for those who could; yet no legal restrictions (including fines) were enforced during 2020 (Orlowski and Goldsmith, 2020). Notably, the Minister of Health and Social Affairs later stated during a parliamentary enquiry that Sweden did in fact have no strategy.

The Swedish Strategy was also influential abroad, and became an argument in other countries including, among others, the United States (US), United Kingdom (UK) and Australia, to loosen restrictions (Orlowski and Goldsmith, 2020; Jung et al., 2020). Supporters of the natural herd-immunity strategy promoted a widespread “controlled” spread in society, to obtain herd-immunity without vaccination (although there was never sufficient evidence for lasting immunity against re-infection) (Jung et al., 2020; Iacobucci, 2020). Sweden, however, did not officially admit that natural herd-immunity was an underlying goal, but the authorities stated that it would be a welcome side-effect or consequence. A large body of internal documents and public statements from various officials during 2020 verify that attainment of herd-immunity was in fact a significant consideration (Lindström, 2021; Nygren and Olofsson, 2021; Orlowski and Goldsmith, 2020; Habib, 2020; Giesecke, 2020; Sörensen, 2020, 2020e; Vogel, 2020; Larsson, 2021). Email conversations and statements from the State Epidemiologist and others show that they at least speculated on the use of children to acquire herd-immunity, while at the same time publicly claiming children played a negligible role in transmission and did not become ill (Vogel, 2020; Bjorklund and Ewing, 2020; Brusselaers et al., 2020).

As a comprehensive evaluation of the COVID-19 policies and implementation in Sweden goes beyond the length and scope of this paper, we focus on the role of scientific evidence for the actions using a narrative approach. In this report, we aim to assess the extent to which Sweden had a pandemic strategy before 2020 and how this strategy was based on science and how it has been implemented and adapted into policy making during the pandemic, focusing on the period until December 31, 2020. We also assess the extent to which scientists, policy makers, and politicians have been involved in the decision-making process. The study is not intended to advance policy or social science theory.

## Methods

Sweden is a monarchy and the largest country among the Nordic countries, bordering Finland, Norway, and Denmark. The Nordic region is a geographical and cultural region in Northern Europe with strong historical, political, and linguistic ties (Mens et al., 2021). The pre-pandemic study setting is described in more detail in Supplement 1, including a summary of the highly decentralised Swedish healthcare system, the socio-cultural, economic, and political structures and ideologies, and the legal framework.

This work follows a narrative structure focusing on the meso-level and macro-level of the Narrative Policy Framework (NPC), i.e., on “how policy actors construct and communicate narratives to influence the policy process”; and “how policy narratives permeate institutions, society, and cultural norms” (Shanahan et al., 2018). This non-experimental design (case-study) evaluates the different c*haracters* in the COVID-19 handling in Sweden, with the Public Health Agency as key player (Shanahan et al., 2018). The *setting* for the meso-level assessment is Sweden during 2020, mainly focusing on the national level, although regional and municipality levels are discussed when relevant. We conducted a content analysis, based on extensive discussions among the co-authors and other national and international experts, a comprehensive systematic literature search of scientific peer-reviewed papers, governmental reports and communications, and mainstream media outlets and digital media (2021g, Shanahan et al., 2018). To also assess how macro-level narratives shaped public policy, (Shanahan et al., 2018) we explored the pre-pandemic shared societal and cultural values present in Sweden.

We evaluated the Swedish COVID-19 policy based on the following four distinct intellectual tasks (Lasswell, 1971; Nachmias, 1979): (a) identification of goals to be achieved in policy implementation, (b) metrics which can be used to assess progress (or lack thereof) with respect to goals, (c) data or evidence related to such metrics: official COVID-19 statistics, reports from healthcare and others and, (d) judgments of responsibility for policy outcomes which might be useful in efforts to improve future performance, to incorporate policy learning into new contexts (Pielke and Boye, 2019). We applied a well-established logical framework of policy evaluation to the intellectual task of assessing the use (and misuse) of science in COVID-19 pandemic management in Sweden. By scientific evidence, in the context of this paper, we refer to the advice of international authorities in infection control (including the World Health Organisation, (European) Centres for Disease Control and Prevention), and the body of peer-reviewed scientific papers. We evaluate the use of scientific evidence in terms of ‘scientific integrity,’ defined as consisting ‘of proper reasoning processes and handling of evidence essential to doing science’ and ‘a respect for the underlying empirical basis of science’ (Douglas and Bour, 2014). We applied the logical structure of a policy evaluation to assess scientific integrity in four contexts: (1) the pandemic preparedness; (2) the evaluation of the different actors in the pandemic; (3) errors and inconsistencies in the recommendations and communications; and (4) the consequences on healthcare and society.

Because of the importance of accurate and reliable sources, we strove to refer to the original source as much as possible, and to peer-reviewed publications—focusing on issues related to the interaction between policy and science. However, there were hundreds of relevant sources including media reports; and consequently, we had to refer to some secondary sources.

A systematic literature search was conducted to identify relevant scientific peer-reviewed papers published on the handling of the pandemic in Sweden and in the other Nordic countries (for reason of comparison) using PubMed/Medline and Web of Science (Supplement 2).

Through the Freedom of Information Laws (Supplement 1), we attempted to gather all email conversations, meeting agendas, meeting notes and press-releases from the relevant parties involved in the decision-making on a national level, with a focus on the Government and Public Health Agency—from 2020 (2020–2021a). An extensive search was conducted to collect published public communications from other relevant parties, including open letters, debate articles, petitions—again focusing on national policy, and the interaction between science and policy.

All authors have lived in Sweden at least through part of the 2020–2021 pandemic and form a multi-disciplinary group with a background in epidemiology, medicine, religious studies, history, political science, and human rights. The group was advised by several national and international independent experts. Ethical approval and informed consents were not applicable since this article does not contain any studies with human participants performed by any of the authors.

## Results

### Pre-COVID: national level of science advisory processes

#### Relevant official Swedish agencies or actors

The most prominent official actors in the pandemic are described in detail in Supplement 3, as well as how they operate and the Swedish crisis management structure. These include: the Swedish Government (including the Prime Minister) and Parliament; The Public Health Agency; The National Board of Health and Welfare; Statistics Sweden; The Swedish Civil Contingencies Agency; The Swedish National Agency for Education; The Swedish Association of Local Authorities and Regions; The Health and Social Care Inspectorate; The Swedish Work Environment Authority; and The Swedish Institute.

#### Health advisory processes including epidemic and pandemic preparedness

Sweden has a well-documented track record of prior epidemics, and corresponding mortality data from mid-eighteenth century and onwards is well-documented by Statistics Sweden (*SCB*) (Ledberg, 2021, 2020m), with four WHO-declared pandemics affecting Sweden since 1900—all with influenza viruses (1918-19 H1N1 “Spanish”; 1957 H2N2 “Asian”; 1969 H3N2 “Hong Kong”; and the 2009 H1N1 so-called “swine flu” influenza) (2019).

The Public Health Agency has published two planning documents in the recent years, one in 2015 to be prepared for pandemic influenza (previous versions in 2009, first version initiated in 2005) (2015) and one in 2019 titled as considering pandemics in general although it also states in the foreword that it is for an influenza pandemic, which is also clear throughout the document, and confirmed by the Public Health Agency in written correspondence (Box 1) (2019).

The purpose of these documents is to serve as background and support for national authorities, the regional infection control physicians, emergency managers and emergency coordinators, and other relevant actors on the regional and municipal levels (2015, 2019). For both plans the central and crucial role of the World Health Organisation (WHO) is highlighted as they will declare the different global phases, disseminate information and to some extent influence the various measures taken (2019, 2015). The International Health Regulations (IHR) are also mentioned, as a legally binding agreement for WHO member countries including “measures for preventing the transnational spread of infectious diseases” (2019).

Box 1: Pandemic preparedness The Swedish strategy published by the Swedish Public Health Agency for “handling a pandemic” is noted to be based on: (2015).

- *A pandemic involves the widespread spread of a completely new type of influenza virus worldwide (…)*.
- *It is not possible to completely stop the spread of infection and eradicate the pandemic virus, neither in the country of origin nor in Sweden. This means that the efforts will focus on delaying the process and limiting the consequences for individuals and society*.
- *Vaccination is the most effective measure to reduce the risk to the individual and prevent the spread of infection. During the early stages of an influenza pandemic, a vaccine is unlikely to be available. A vaccine takes at least 3-6 months to develop*.
- *Before a vaccine is available, antiviral drugs will play a major role in reducing the risk to the individual and delaying the course of the pandemic*.
- *Once the vaccine is available, antiviral drugs are still important to reduce the risk of serious illness*.

The overarching aim according to the 2019 pandemic plan was:

- *to minimise mortality and morbidity in the population*
- *to minimise other negative consequences for individuals and society*.

The 2019 plan further states that the goals are:

- *Public health must be affected as little as possible*.
- *The negative effects on society must be as small as possible*.
- *Confidence in the authorities, healthcare and care must be maintained*.

In both planning documents, the distribution of information to the infection control physicians, the National Board of Health and Welfare, the ECDC and WHO is stressed.

The collaboration on national, regional and community level include: (2019).

- *collect and share information to get a common picture*
- *discuss risk assessments and measures*
- *coordinate measures*
- *coordinate and communicate messages*

All Swedish crisis management is also based on three underlying principles: (2019).

1. Responsibility (*Ansvarsprincipen*) Anyone who is responsible for an activity under normal conditions has a corresponding responsibility during a crisis. This also includes initiating and conducting cross-sectoral collaboration
2. Equality (*Likhetsprincipen*) The activities in the event of a crisis must resemble the normal as far as possible
3. Proximity (*Närhetsprincipen*) A crisis must be handled where it occurs and by those most directly affected and responsible.

The different actors and their roles are described, and a checklist was presented for the different phases of the pandemic. Some simulations and scenarios (targeted towards influenza) are presented, and the proposed strategies rely on contact tracing (first 1000 cases), antiviral drugs and vaccination (for risk groups only or total population) (2019). Yet, as described in Supplement 3, none of the actors have an exclusive focus on infection control.

### COVID-19: How the pandemic was handled and evolved in Sweden

**Fig. 1:**
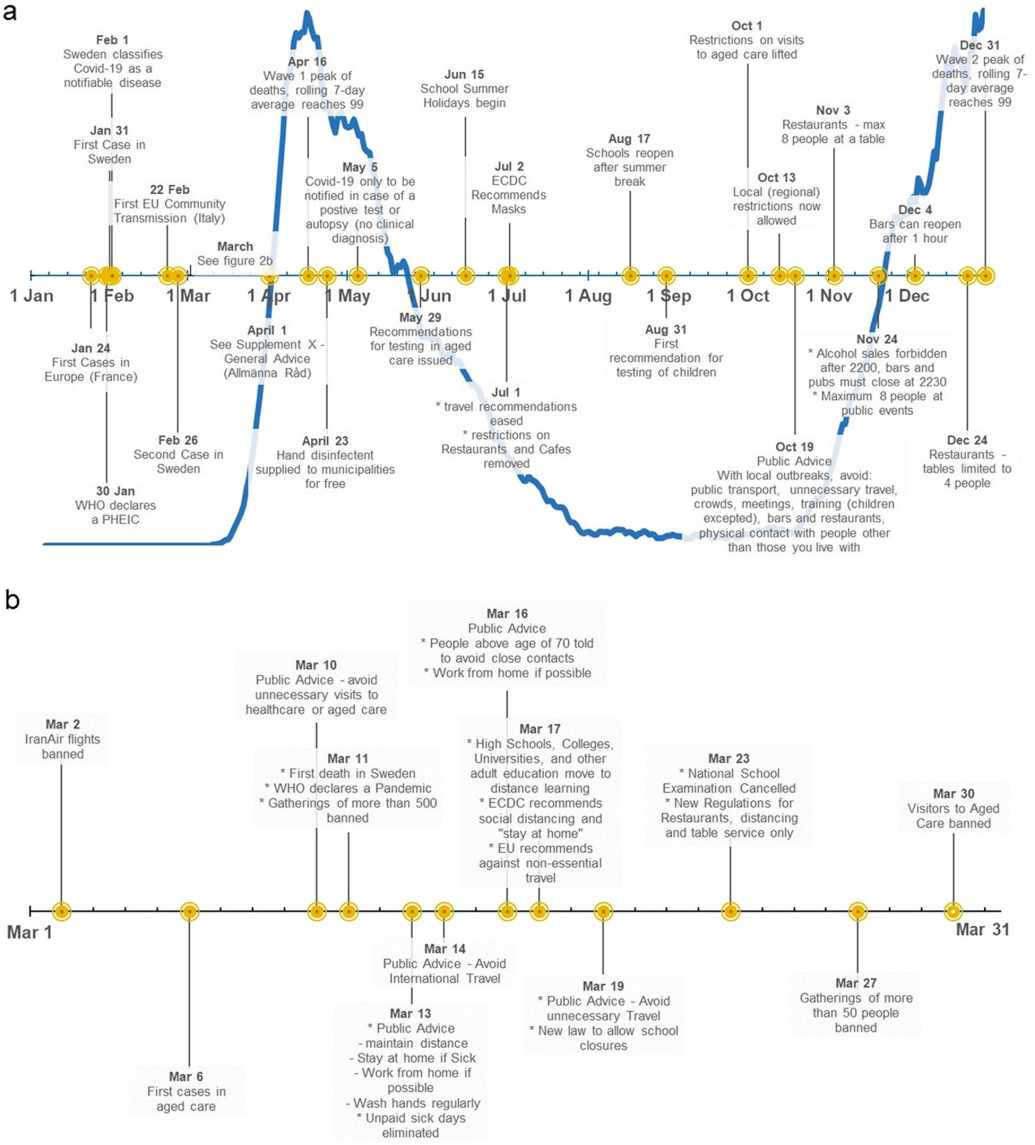
Timeline. **a**—Swedish Pandemic Timeline. Key Events 2020. **b**—Swedish Pandemic Timeline. Key Events March 2020.

The early global, European, and Swedish timelines are presented in detail in Supplement 4 and Fig. 1.

#### The Swedish strategy: coordinated by the Public Health Agency

The Swedish pandemic response corresponds to a mitigation strategy keeping large parts of the society open, strongly relying on individual responsibility (Nygren and Olofsson, 2020). This strategy was implemented by the Government based on advice from the Public Health Agency (Mens et al., 2021; Kavaliunas et al., 2020; Nilson, 2021). Sweden’s legal framework is given as one of the justifications to legitimise a passive and delayed action in Sweden—characterised by less strict restraints than most other European countries. “Lockdowns”, strict “stay at home” orders (or even advice) were never implemented (Mens et al., 2021; Kavaliunas et al., 2020; Nilson, 2021). The Swedish constitution states that “Swedish citizens have the right to move freely within Sweden and leave the country”, and have the right to receive education (Mens et al., 2021; Wenander, 2021). While the Swedish Communicable Diseases Act can restrict individuals and the Public Health Agency has the right to quarantine geographical areas, the Government and other officials claimed that a general lockdown was not legal (Mens et al., 2021). Consequently, the Swedish strategy was based primarily on non-binding “soft-law” recommendations, not compulsory nor enforced like in other countries (Mens et al., 2021; Wenander, 2021, TT, 2020a).

The Public Health Agency recommended early on “to avoid unnecessary travelling and social events, to keep distance to others, and to stay at home” if symptomatic, and especially to wash your hands regularly. In addition, those above age 70 were “advised to avoid social contact” and visitors to retirement homes were banned on March 31, 2020 (2020i, Tegnell, 2021; Möller Berg, 2020). Upper-secondary schools and universities went into remote learning on March 17 until August 2020; and again in December 2020 (Tegnell, 2021). In general, schools for children under 16 remained open besides some local and temporary closures related to spreading infections (in the school or community), based on decisions of the individual schools. School attendance is compulsory in Sweden and no exception was made for children with parents in risk groups or, initially, for children with family members with confirmed COVID-19. Measures to prevent infections were not widely implemented or mandated to the same extent as in other countries (with local exceptions)(Supplement 4) (Bjorklund and Ewing, 2020).

#### The role and actions of the Government

The Government’s stated overarching goal with regard to the pandemic is to safeguard people’s lives and health and to secure the healthcare capacity.(2020–2021 (regularly updated)) The Government stated that their policy and decisions aim to: (i) limit the spread of infection in the country; (ii) ensure healthcare resources are available; (iii) limit the impact on critical services; (iv) alleviate the impact on people and companies; (v) ease concern, for example by providing information; and (vi) ensure that the right measures are taken at the right time.(2020–2021 (regularly updated)) Yet, these aims were merely “talking points” since they were not backed up with specific actions or plans. Minister of Health and Social Affairs Lena Hallengren publicly stated in the Parliament’s interrogation that the Government strategy was not to have a strategy, and to impose the right measures at the right time (Larsson, 2021).

In non-crisis situations, the Government is not supposed to influence the work of individual Government agencies such as the Public Health Agency, especially in regard of individual citizens (Mens et al., 2021; Wenander, 2021). In theory, crisis management on the governmental level was supposed to be coordinated by the Prime Minister’s Office (or Ministry of Justice as delegated by the Prime Minister), calling meetings with the relevant ministries (e.g., health, education, justice) and then activating the crisis management group. Then, the different agencies/authorities were supposed to collect information and act. These agencies are not supposed to govern themselves, yet they have operative responsibility—and it is in this respect that they have autonomy (Wenander, 2021). Yet, during the pandemic, the crisis management function in the Ministries (and Prime Minister’s Office) again did not work (see Supplement 3, previous disasters). A large part of the responsibility was delegated to one agency (the Public Health Agency), and the government failed to seek information themselves (2021f, Jerneck, 2021; Nordberg and Mattsson, 2020; Kleja, 2020). In addition, the Public Health Agency has no responsibility for the range of issues (according to its directives—*Myndighetsinstruktion*), and therefore it seemed able to evade accountability.

During 2020, the Swedish Government, the Prime Minister and Minister of Health and Social Affairs, mainly referred to the authority of the Public Health Agency and the regions/municipalities. The Prime Minster rarely gave interviews and only few pre-recorded messages—and no crisis group was formed in the Parliament (Sörensen, 2020). Both the Prime Minister and Minister of Health and Social Affairs publicly declared they had no competence considering pandemics or medical issues. In effect the democratic institutions ceased to function (Sörensen, 2020). The Public Health Agency strived for and asked early for an overarching impact on the decision-making, which was granted by the government (Kleja, 2020). The Agency managed in this way to control the total decision-making of the government. Citizens and the public were never fully informed or included in the deeper reasoning behind their decisions (Kleja, 2020).

After the Parliament made temporary amendments to the Communicable Diseases Act (*Smittskyddslagen*) the Government and The Public Health Agency had the legal means to impose stronger measures, such as closing restaurants, shopping malls or airports (Supplement 4). In addition, measures and actions in schools, health- and elderly care, such as masking or testing, could have been recommended or mandated without any legal restrictions. There could also have been more measures to simplify and increase distance-work, limit the spread of infections by mandating (or at least recommending) face masks and implementing the legally required test-and-trace strategy (Supplement 5), provide care for the elderly (Supplement 6) or by allowing children and adults to protect themselves if they (or their family members) belonged to risk groups (instead of forcing them to attend school in person)(Supplement 7). The argument that stronger restrictions were not legal or State-organisationally impossible in Sweden is not valid for this (inter)national crisis situation (Wenander, 2021; Nordberg and Mattsson, 2020). Accountability for this apparent lack of crisis management remains unclear (Wenander, 2021, Nordberg and Mattsson, 2020).

During the first year and a half of the pandemic, there was never any strong political opposition against the pandemic actions of the Government. Only the Swedish Democrats (populist nationalistic right-wing) were slightly critical of the Swedish strategy in late-Winter/Spring 2020 and raised some questions in the Parliament (Sörensen, 2020). Most other parties did not oppose or question the strategy, although there was some questioning around May 2020 when the deaths were accumulating, and Sweden was among the worst in Europe considering the daily mortality rate/million (in the global top 10 by April 30, 2020)—while the Swedish economy was also heavily impacted.(Sörensen, 2020; Johansson et al., 2021) This may have helped to finally implement increased testing in Sweden in June 2020 (Supplement 5).

The precise responsibility of the different Government agencies (including the National Board of Health and Welfare and the Swedish Civil Contingencies Agency) remained unclear for the public, since none stepped up to take responsibility. Both the Government and the Public Health Agency declined any responsibility for the situation in the elderly homes and referred to the responsibility of the municipalities (responsible for nursing homes) and regions (responsible for equipment, crisis preparedness, and healthcare—especially in elderly care) (Supplement 6) (Sörensen, 2020). The care homes and hospitals referred back to the Public Health Agency since that agency provided advice on personal protective equipment and routines to follow (including face masks) (Sörensen, 2020).

#### The Swedish pandemic response plans

There has only been one official crisis management plan released during the period of the pandemic, relating to the planned handling and actions—which we obtained through Freedom of Information laws (although some parts have been censored).(2020g, 2020h, 2020e). This plan was issued by the Ministry of Justice in June 2020, updated in September 2020, and focused on the impact of the pandemic on society (2020g, 2020h). The key points included: not to spread fear and panic, to prevent social unrest, and to limit the impact on the industry/economy/hospitality sector. This plan does not include anything about healthcare, healthcare capacity or infection control measures (2020g, 2020h).

#### Actions in Swedish healthcare (responsibility of regions) and face masks

To meet the needs for both COVID-19 related healthcare needs and other healthcare, this pandemic has put an enormous strain on the global healthcare supply (Bark, 2020). There has most likely been a before—and within hospital triage of individuals with potential COVID-19 in several places in Sweden. During spring 2020, many individuals were not admitted to the hospitals, and did not even receive a health examination since they were not considered at risk—resulting in individuals dying at home despite trying to seek help (Vogel, 2020; Bjorklund and Ewing, 2020; Hiselius and Arnfalk, 2021). In addition, there were triage instructions available in Stockholm region, showing that individuals with comorbidities, body mass index above 40 kg/m^2^, older age (80+) were not to be admitted to intensive care units, since “they were unlikely to recover” (Vogel, 2020; Söderberg et al., 2020). Although it has been disputed that these were implemented in practice, the age distribution of the admission at the ICUs strongly suggests a selection bias for admission to the ICU based on age (Kamerlin and Kasson, 2020; Funck, 2020, 2020o; Strålin et al., 2021). Despite worrying signals from different hospitals over-stretching their limits, the Public Health Agency and Government kept claiming there were still ICU beds available in Sweden, and that their strategy did not fail since they were able to keep the contagion at levels that the healthcare system could handle (Sennarö and Zachrisson, 2020; Rolander, 2020; Bark, 2020; Bjorklund and Ewing, 2020). Nevertheless, Sweden scored lowest on accessibility of intensive care beds based on a study of 14 European countries looking at the impact on COVID-19 case fatality ratio, with spatial accessibility being relatively high near some population centres, but lower in rural area (Supplement 6) (Bauer et al., 2020).

The largest Swedish trade union (*Kommunal*), which organises 500,000 workers on the municipal and county levels, questioned the Public Health Agency’s claims that proper face masks or personal protective equipment were not needed when treating COVID-19 infected patients—and went on local strike in April 2020.(Sörensen, 2020; 2021a). They used the Swedish work environment law to advocate for protecting their workers (not the elderly living in the care home), and the Work Environment Authority ruled in favour of Kommunal (2021a). Yet, due to lobbying by the Swedish Municipalities and Regions, this decision was reversed (Sörensen, 2020, 2021a). To note: face masks and basic equipment were lacking and could not be provided to all personnel (Sörensen, 2020). The Swedish Public Health Agency finally recommended face masks or protection in hospitals and care homes on June 25, 2020 (after >5000 deaths)—yet even then, their recommendation to use face masks was only for treating confirmed or suspected COVID-19 cases—while visors/face shields were more commonly used (Sörensen, 2020), although cited as not sufficiently effective against airborne infections. The Public Health Agency and Ministry of Health and Social Affairs discouraged the use of face masks by the public and claimed face masks are ineffective, dangerous and spread fear (2020a, Vogel, 2020; Bjorklund and Ewing, 2020, 2021j). Although some healthcare institutions did implement mask-use on their own initiative, mask wearing was actively discouraged or “not allowed” (at least at some points during the pandemic) in healthcare settings, elderly homes, schools and other settings, even resulting in professionals being laid off and people being denied access (Lundquist, 2020; Orange, 2021; Nordwall and Bolin, 2021; Ågren, 2021; Vogel, 2020; Bjorklund and Ewing, 2020, 2021j).

#### The elderly care (responsibility of municipalities)

Another heavily criticised part of the Swedish approach was prevention control and management of the infected elderly, both in elderly care facilities and home-care (including insufficient personal protective equipment) (Nilsson et al., 2021; Strang et al., 2020; Ohlin, 2020; Möller Berg, 2020; Höglund, 2020). The decision to provide end-of-life care to many older adults is highly questionable; very few elderly have been hospitalised for COVID-19. Appropriate (potentially life-saving) treatment was withheld without medical examination, and without informing the patient or his/her family or asking permission (Supplement 6) (Habib, 2020; Sörensen, 2020; Ohlin, 2020; Möller Berg, 2020). Many officials kept denying any responsibility (Falck, 2021; Möller Berg, 2020; Sennarö and Zachrisson, 2020), and there was only limited public outcry in Sweden when this came out, the common narrative being that those in care homes are expected to die soon anyway (see part on ageism in Swedish society, Supplement 1).

#### Children and schools

Children were also majorly affected by this pandemic, since the Swedish strategy was strongly against any school closures or measures to protect children, as clearly communicated by the Public Health Agency, the Minister of Education and others (Supplement 7) (Höög and Adman, 2020; Nilsson, 2020; Delin and Mahmoud, 2020). Testing has also been restricted and often impossible for children especially if asymptomatic, so no reliable numbers are available (Vogel, 2020). Nevertheless, many children are still suffering from serious long-COVID, more have lost one or two parents, and several children died—as also noted in the investigation report of the children’s ombudsman (*Barnombudsman*) (2021b, Törnwall, 2020, Bjurwald, 2021).

In-school attendance in Sweden is compulsory (*Skolplikt)*, thus distance learning or home-schooling is not allowed in Sweden (Lindblad et al., 2021). Even during the pandemic, no exceptions were made for children with risk factors, or parents who were clearly at risk for serious COVID-19 infections. Schools and municipalities have alerted social services and parents who wanted to protect their children by keeping them at home were fined. Few or no infection control measures were taken in many schools, and face masks were often not allowed (Aschwanden, 2021; Höög and Adman, 2020). Legally this is in conflict with the Parental Code (*Föräldrabalken*), which states that parents should protect and provide care for their children, and with the United Nations Convention on the Rights of the Child (*Barnkonventionen*)(2021k), which Sweden has adopted. Most schools remained open even during outbreaks, since this was the advice given by the authorities (Höög and Adman, 2020, 2021b).

The Public Health Agency denied or downgraded the fact that children could be infectious, develop severe disease, or drive the spread of the infection in the population; while their internal emails indicate their aim to use children to spread the infection in society (Lindblad et al., 2021, 2020–2021b; Höög and Adman, 2020; Vogel, 2021; Ludvigsson, 2020).

#### Lack of transparency

This *evasive accountability structure* was even more complicated by the practice of secrecy and lack of transparency (Sörensen, 2020). Even the number of available ICU beds per region was not publicly available, and regions were unwilling to share information on the spread of the infections to the municipalities (even leading to legal action) (Sörensen, 2020; Cederberg, 2021). Many schools did not inform parents or even teachers about confirmed COVID-19 transmission on the premises, nor reported it to official agencies, and urged parents not to tell if their children were infected—since this would “spread fear” (Hedman, 2021; Besançon et al., 2021; Höög and Adman, 2020, 2021b). Some municipalities refused to declare the number of deaths in the care homes and there was an attempt to keep the death rates “covered up” at a regional level (Sörensen, 2020). Even an outbreak of COVID-19 on a maternity ward in the Uppsala University hospital was initially kept secret (Sörensen, 2020).

One of the officials of the Swedish Civil Contingencies Agency (Mikael Tofvesson, Head of the Information Protection Unit, Swedish Civil Contingencies Agency) even said that truth can be disinformation if it affects public trust in the authorities (Lundgren and Tofvesson, 2021).

Despite the Swedish Freedom of Information Laws (Supplement 1) (2020e), several requested emails, meeting minutes or even agendas of meetings were not obtained, or only with large parts of the text redacted. For example, the underlying models for decision-making from the Public Health Agency and their assumptions were not made public—particularly not during the first months of the pandemic (they finally published some of the coding on April 24, 2020) (Sörensen, 2020). Therefore, even expert scientists could not evaluate these and had to rely on press conferences and interviews given by Anders Tegnell and others (Sörensen, 2020).

Although Sweden is one of the few countries with multiple high-quality health and population registries, there were important problems with the reporting of COVID-cases, hospital admissions and even deaths. This was not only due to limited testing, but also to case-definitions being relatively strict and being changed several times during the pandemic (Villani et al., 2020, 2020f, 2020b). There were delays of several weeks, and differences between the official sources (Public Health Agency, National Board of Health and Welfare, Statistics Sweden) (Bjorklund and Ewing, 2020). There are also concerns about data-manipulations, in particular of COVID cases and child deaths (Vogel, 2021; Bjorklund and Ewing, 2020).

This pattern of secrecy, cover-ups and data-manipulations was present on national, regional and municipal levels—with even official websites (e.g., Government strategy) being updated without changing the “date of last update” (Sörensen, 2020). For example, although several of the people involved publicly made statements that face masks were not needed, or even “dangerous” or contra-productive (2020r, Bjorklund and Ewing, 2020)—they later claimed they had always been supportive of their use (Tegnell, 2021, 2021j). The Swedish Work Environment Authority and the State Epidemiologist even started erasing related emails requested by journalists.(Sörensen, 2020; Sandberg, 2020). Although this is illegal, the practice of withholding information and erasing emails became widespread among official agencies during the pandemic—leading to a so-called “shadow management”, since apparently the risk for legal sanctions is very low for power holders (Sörensen, 2020; Sandberg, 2020).

## Newly created (or modified) science advisory processes during COVID-19

### Formal science advisory processes

#### Public Health Agency: expert committee

After receiving critique that the Public Health Agency did not consider knowledge or expertise outside the agency, Director General Johan Carlson, announced the members of a newly formed advisory group on April 17, 2020. This group included six clinicians and scientists with expertise in clinical microbiology, clinical virology and infection control (2020d, Trysell, 2020). None of the academic experts that had spoken up publicly with approaches more in line with WHO were invited or selected. There were no notes taken from this group’s meetings and in an interview in August 2020, Dr Hans Fredlund, an infection control physician from the group, explained that they did not discuss large, overall issues, something he had thought they would.(Trysell, 2020). “If the group of experts were to have any real function they probably should discuss questions of principles as well,” he said (Trysell, 2020).

The *National Board of Health and Welfare* and *Statistics Sweden* have released occasional press-releases and data on, e.g., excess mortality, yet to our knowledge, did not play an official scientific advisory role directed towards the Public Health Agency or Government.

#### The Corona Commission and the Swedish Health and Social Care Inspectorate (*IVO*)

On June 30, 2020, the Swedish Government appointed a Corona Commission (*Coronakommissionen)* to evaluate the measures taken to limit the spread of COVID-19 in Sweden. This group has been commissioned to evaluate the measures taken by the government, the relevant administrative authorities, the regions and the municipalities to limit the spread of the virus, and the effects of such a spread (2021c). The Commission will also compare this with other *relevant* countries (2021c). A full Government reckoning of the handling of the pandemic won’t be made public until February 2022, but an interim report on the spread of the virus in nursing homes was released on December 15, 2020, with a second interim report expected end of October 2021 (2021c, 2020l). The first interim report noted that Government measures were late and inadequate, and called the spread of the virus in society the “single most important factor behind the major outbreaks and the high number of deaths in residential care ”(2020q). The Commission found that “the strategy failed to protect the elderly” (Supplement 6) and that the Government’s measures were both insufficient and late, and noted the late onset of widespread testing (2021c, Bjorklund, 2020).

The Swedish Health and Social Care Inspectorate (*IVO*), a government agency supervising healthcare and social services, reported widespread, “systemic” failures in its report released November 24, 2020 (Supplement 6) (2020q). Its investigation cited “serious shortcomings” including that one-fifth of the people in nursing homes were denied their right to receive an individual medical assessment (2020q, 2020n). Less than one-tenth of COVID-19 patients had been examined by a physician. Some patients were put on end-of-life treatment without a positive test and failures were cited in all of Sweden’s 21 healthcare regions (2020q). Despite this, *IVO* further found that none of the regions had taken responsibility for the poor treatment of infected nursing home residents. Prime Minister Löfven said on September 8, 2020 that “The casualties in Sweden are mostly in elderly homes and older people (…) That has nothing to do with people walking in the city” (Bjorklund and Ewing, 2020).

#### Life science/medicine faculties of universities

On February 27, 2020, the Vice Chancellor of Sweden’s leading medical research institution in Stockholm, Karolinska Institute (KI), Ole Petter Ottersen appointed a special Karolinska Institute expert group for the COVID-19 outbreak (Ottersen, 2020). Yet, this selected group had clear ties with the Public Health Agency, leading to questionable independence (Supplement 8). Hence the Swedish public was led to believe that several experts had separately come to the same conclusion that the unique Swedish strategy was the right.

The other major Swedish universities were less visible in the media on the Swedish handling of the pandemic, yet several individual academic researchers questioned the Swedish strategy in the social, (inter)national media, and scientific literature.(Brusselaers et al., 2020; Claeson and Hanson, 2020; Claeson and Hanson, 2021; Ewing, 2020; King et al., 2020; Lindahl et al., 2020; Naguib et al., 2020; Nkengasong et al., 2020; Lindström, 2020b; Sörensen, 2020; Bjurwald et al., 2021). Many have been reprimanded by their superiors, e.g., that they were supposedly not allowed to use their university affiliation, or that they were criticised for undermining the authorities—clearly breaching the right of (Academic) Freedom of Speech (1948, Vrielink et al., 2010; Vogel, 2020).

### Informal science advisory processes (without official mandate)

Several Swedish experts warned about the pandemic already in January 2020—but this was dismissed by the authorities and even ridiculed in the media (Sörensen, 2020; Claeson and Hanson, 2021, 2021e). From the start of the pandemic, there was critique from within the medical and scientific community—stressing the lack of preparedness of healthcare and society.

Yet, from early on, the Swedish strategy seemed to have a general and widespread support at all levels of the population. The Public Health Agency and supporters of the Swedish strategy also actively discredited any critique and national/international scientific evidence, cherry picking papers or parts of papers disregarding the larger amount of evidence suggesting the opposite (Sörensen, 2020; Brusselaers et al., 2020).

Questioning the strategy, even in academic settings, the media, or the Government, was apparently not accepted by the Swedish society and considered disloyal, and critics were discredited as “hobby-epidemiologists” or lacking competence (Steineck et al., 2021; Bjurwald et al., 2021).

As a result, scientific debate appears to have only occurred by means of newspaper opinion pieces, without direct discussions or debates between the relevant parties.

To give a few examples: (Bjurwald et al., 2021).

On March 3, 2020, Fredrik Elgh, an internationally esteemed Professor in clinical virology, and former superior of Anders Tegnell (during 2000–2002 at the Institute for Infection Control), wrote about his past experience with pandemics and urged Sweden to prepare (Elgh, 2020; Majlard, 2020b). Fredrik Elgh was heckled and compared to a Sami (indigenous Swede) “who tracks the future in fish stomachs” by Johan Carlson, Director General of the Public Health Agency, and scorned by others (Majlard, 2020b).

On March 13, 2020, an editor of one of the largest Swedish newspapers urged Sweden to close down to protect the country (Woldarski, 2020). Johan von Schreeb, a professor in disaster medicine and part of the Karolinska Institute COVID advisory group, which worked closely with the Public Health Agency, responded that the criticisms against the Public Health Agency/Tegnell are “indecent”, and that this editor was “undermining Swedish expertise” (Von Schreeb, 2020; Brusselaers et al., 2020).

Johan Carlson called criticism against Anders Tegnell and the Public Health Agency “unworthy” (Nordlund, 2020). From early March, similar communication likely contributed to the glorification of Anders Tegnell as Swedish hero and idol (Corcoran, 2020; Bjurwald et al., 2021).

#### The Royal Swedish Academy of Sciences (Kungliga Vetenskapsakademien)

The Swedish Royal Academy of Sciences is an independent organisation whose overall objective is to promote the sciences and strengthen their influence in society. This organisation did not have any direct role, or visible impact on, the activities of the Swedish Public Health Agency. The Academy provided information for public debate, yet there have been no formal discussions with the Public Health Agency.

At first, the Swedish Royal Academy appointed a working group led by Göran Hansson (Permanent Secretary of the Academy) and then later an expert group (2020s). On May 8, 2020 with minor revisions in June and August, the Academy working group produced a report and updates, with title, “Facts and debate about COVID-19,” on the nature of the COVID-19 virus and the Swedish response to it in Sweden (2020j). Most of the discussion was on the science to understand the virus. The revision in June was to add the recommendation of face masks to help limit disease spread (Supplement 8) (2020j).

Much of the advice from the Royal Academy of Sciences was not followed by the Public Health Agency or was later only partially implemented. The expert group did not explicitly deal with air filtration, schools; and virus variants were not of concern at that time—although some predictions had been made.

#### Science Forum COVID-19 (*Vetenskapsforum* COVID-19)

The Swedish Science Forum COVID-19 is a non-profit organisation officially founded in June 2020, with currently approximately 40 independent physicians and scientists with a wide range of backgrounds (e.g., virology, epidemiology, mathematical modelling, chemistry, physics, societal issues, global politics, ethics), many with a notable (inter-)national track record (Sörbring, 2021). The declared mission of the association is to save lives and help prevent all forms of suffering during the COVID-19 pandemic by educating the Swedish public about ongoing scientific discussions through (pro-bono) debate articles, television and radio interviews, social media presence as well as live and recorded discussions with guests (Supplement 8).

The articles treated topics like asymptomatic spread of infection, aerosol transmission, face masks, the school situation, child transmission and other topics where Sweden remained an outlier and not officially aligning to international scientific consensus (2021l).

#### Other informal science advisory processes outside the bounds of the playbook or newly created mechanisms

In early April, >2000 scientists in Sweden signed an open letter urging the Government to take stronger action (Abadi and Leslie, 2020). In addition, several groups have formed on social media including groups of physicians with “long-COVID”, a group advocating for stricter measurements in schools to protect children, another group advocating for correct information in the national and international media about the Swedish strategy.

The Swedish Medical Journal (*Läkartidningen*) also published debate articles and opinion pieces, yet these mainly supported the narrative of the Public Health Agency.

The Swedish Journal of Political Science (*Statsvetenskaplig tidskrift*) has also published several articles including a Special edition on the “Corona pandemic—decision-making in difficult conditions”.

In addition, there were also individual researchers strongly supporting the Swedish strategy, with questionable independence because of the close contact with the Swedish Public Health Agency or other ties to authorities—yet they were regularly given a bigger stage in the Swedish mainstream media although often clearly spreading disinformation (Bjurwald et al., 2021, 2021e),

## Comparison of implementation of science advice under COVID-19 with the “playbook”

### Was the playbook followed?

Prior to 2020, there were only pandemic planning documents for Influenza pandemics, and these were insufficiently adapted to this type of virus (*Corona*). The mindset that influenza cannot be eradicated and stopped completely appears to be a key element of the Swedish strategy. Testing-and-tracing was never fully implemented (Supplement 5), neither was quarantine/isolation or school closures for younger children, all mentioned in the pandemic plan.

During the first few months, there was little official and documented communication between the different official actors. The Public Health Agency provided press-releases and held press conferences with representatives from the Swedish Civil Contingencies Agency and National Board of Health and Welfare and other officials (Bjorklund and Ewing, 2020). Yet barely any data or communications were made public, and the few critical questions of the media at the press conferences were mainly ignored (Bjurwald et al., 2021; Lindström, 2021). Because of this sparsity of data, including records of meetings, it appears the strategy was based on the opinions of a very limited number of individuals (primarily Anders Tegnell, Johan Giesecke and Johan Carlson at the Public Health Agency). This small group of “experts”, with a narrow disciplinary focus, also went beyond their mandate and expertise—for example, commenting on the economic effects—and demanding more power/authority than they were legally allowed to have (Kleja, 2020). On June 3, 2020 Anders Tegnell admitted that Sweden could have done more in this pandemic.(Wise, 2020). Yet, after international media attention, he retracted this statement later that day and confirmed Sweden followed “the right strategy” (Wise, 2020).

The political system relied on these opinions, claiming they did not have any expertise themselves—and many regions/municipalities just copied these advice/opinions——yet were then afterwards “blamed” for mishandling the pandemic.

The pandemic planning documents had a major focus on the exchange of data and recommendations with the WHO and ECDC, yet Sweden clearly went against their own recommendations from the start (2021e). No functioning advisory committee with a multi-disciplinary group of scientists was formed—and the debate on the Swedish strategy mainly took place in the mainstream and social media; besides back-door communication with a “selected few”, strongly supporting the Swedish strategy from the start, as described above.

### How was it modified?

Officially, the pandemic plan was not modified, nor was it clearly communicated. The strategy communicated to the public included some talking points, but these were not sufficiently linked to concrete actions. Mounting evidence (including on aerosol spread, asymptomatic and presymtomatic spread, the effectiveness of face masks, infections in children) was ignored, and contradicting and misleading information was spread to the public (2021h, 2021j).

### Were expectations of science advice realised?

There was no official, democratic, or multi-disciplinary science advice during the pandemic. The Swedish strategy has been going against the international consensus (including WHO, ECDC, CDC) from the start. Several things which have been considered proven, or at least most probable by the international scientific community, have been or are still denied by the Swedish authorities. This includes asymptomatic and pre-symptomatic spread, airborne spread (and not fomites as the most common route of infection), the importance of testing and tracing, the efficacy of face masks, the waning immunity after COVID-19 infection and possibility for re-infection, the role of schools and children in the spread of infections, symptomatic and infectious children (and children who die), and “long-COVID”. The precautionary principle followed by most countries, was not followed, since officials even said symptomatic individuals could go to work and pick up their children at school. The Public Health Agency also downplayed the severity of the pandemic and community spread in Sweden—claiming repeatedly in the media that COVID-19 would never spread in Sweden, that the number of infections was decreasing (despite evidence to the contrary), that COVID-19 was not a bigger threat than previous corona virus infections and influenza, that natural herd-immunity was within close reach, and that there would never be a second/third/fourth wave (Brusselaers et al., 2020; Vogel, 2020; Bjorklund and Ewing, 2020).

### Was it a deliberate strategy?

It could be questioned to which extent the Swedish handling of the pandemic was a real strategy, or a consequence of the lack of a coordination, communication, and debate between all relevant parties—with especially the Government being absent from the scene. The Swedish pandemic handling was completely dominated by the Public Health Agency. We argue that this is a drastic deviation from 280 years of respect for scientific facts and trusting cooperation between politicians, civil servants, and the research community. The last time the leading figures in the research community were assisting the government was in the response to the HIV pandemic.(Andréasson et al., 1997).

Concerning COVID-19, the Government never effectively implemented measures to achieve the strategy aims they outlined. There were numerous conflicting statements by public officials, contradicting each other or what they said themselves previously. For example, representatives from the Public Health Agency and Government (including the State Epidemiologist and Prime Minister) stated that the Swedish strategy was not different from the strategy in other countries (Tegnell, 2021; Sennarö and Zachrisson, 2020) yet Sweden was also the only country having the right strategy and “all other countries were wrong” or experimenting (2020a, Majlard, 2020a; Johansson et al., 2021). No strategic documents considering healthcare and infection control, or solid meeting notes of any initial meetings between the Government and Public Health Agency were made publicly available. These could not be obtained when requested, since apparently there were few meetings, and if these took place, no meeting notes were recorded. The assumptions and modelling from the Public Health Agency were not communicated, or they were presented in a methodologically unsound and unscientific manner raising more questions.

Even after national and international criticism and condemning official assessments of the “failed” Swedish strategy by different (international) committees and working groups, no drastic changes occurred, scientific evidence was still ignored, and the strategy was still heavily promoted (Ludvigsson et al., 2020, 2020a). There is still no open, democratic platform for decision and policy making; nor changes in responsibilities at the Public Health Agency or Government because of their inaction or suboptimal and unscientific/unprofessional functioning.

## Characterisation of the roles played by official and informal scientific advisors and mechanisms

The Swedish paradigm to handle COVID-19 was evidently different from the majority of countries, failing to follow international advice of the WHO/ECDC or scientific evidence.

Categorising the roles of scientists using Roger Pielke’s Honest Broker framework(Pielke, 2007), it is clear there have been few people officially involved. The Government has relied entirely on the Public Health Agency and delegated operative responsibility to them. Other official organisations (including health- and elderly care, schools) have also relied on this agency without assessing scientific evidence or other expert advice themselves.

Many individuals in the above-mentioned informal science advisory section could be described as *Pure Scientists* within the Honest Broker framework, including Science Forum COVID-19 and The Royal Swedish Academy of Sciences. With limited experience considering policy making prior to the pandemic, these groups primarily focused on presenting facts and scientific evidence. Although several members of these groups have attempted to engage in dialogue with decision-makers, these actions were mostly unsuccessful. The Public Health Agency was created with the goal of this role being sourced outside of the Agency, however, as outlined in this paper this does not appear to have occurred during this pandemic.

The Public Health Agency, and primarily Anders Tegnell, have been positioned as “*Science Arbiter*” during the pandemic by both the Swedish Government and media. As the “expert authority” the Agency has been trusted to evaluate and interpret prior and new science relevant to guiding the pandemic response. In practice, when questioned on scientific matters only specific factual questions posed by the Government or media are answered, with replies often not based on a complete picture of the available evidence or on any data at all. Critical questions have usually been avoided at press conferences. Other groups, such as the Royal Swedish Academy of Sciences have also attempted to fulfil this role but have mostly been side-lined.

In practice, the Public Health Agency has primarily acted as an *Issue Advocate*, reducing the scope of choice available to decision-makers, actively discouraging discussion of alternative options, and instead focusing on promoting and justifying their own policy choices. Their narrative at regular press conferences was presented by the national media with little critical questioning (despite regular contradictions), or fact checking.

Finally, in the framework, we have the *Honest Broker of Policy Options* who seeks to expand, or at least clarify, the scope of choices available to decision-makers. This role has not been taken by any official player in this debate, nor have any unofficial candidates been apparent. Several individuals have tried to influence the public and decision-makers through traditional and social media and scientific papers, but with limited success.

## Evaluation of the contributions and roles of science advice in the context of national public health decision-making and outcomes

### Science advice and national public health

Several errors seem to have occurred (also Supplement 9):

1. In terms of governance, the whole Government crisis management function ceased to operate independently, and all decision-making landed on the Public Health Agency. This threatens *parliamentarian democracy* since no discussion seemed to have taken place in any political party on the pandemic response and the share of responsibility in the pandemic. Since all parties have agreed not to turn virus politics into an issue, citizens cannot affect the policy by their vote in the next election.
2. The Public Health Agency was systematically *incorrect in their risk assessments*, and *ignored scientific evidence* on suppression strategies, airborne transmission, pre-symptomatic and asymptomatic spread, face masks, children and COVID-19, “long-COVID” (Steineck et al., 2021; Pashakhanlou, 2021; Ludvigsson et al., 2020; Bjurwald et al., 2021; Delin and Mahmoud, 2020)—and insufficiently implemented and adapted their pandemic response plan, which was constructed for an influenza pandemic. It seems misinformation or incomplete information were communicated deliberately by the authorities to the public, facilitating the spread of the virus in the society (2021h). Although the Public Health Agency took an autocratic lead in the handling of the pandemic, this agency lacks competence in politics, economy, social and behavioural sciences, ethics, and others—competences, which were not complemented sufficiently elsewhere.
3. The *‘Precautionary principle’* which is in fact written into the EU’s function, has been ignored, since a “wait-and-see” passive approach has been followed. Sweden never aimed at suppressing transmission of infection; only to not overwhelm healthcare—contrary to the advice of WHO and ECDC.
4. *International scientific advice was ignored and discredited* on almost all levels—since the only advice communicated or adhered to was issued by the Public Health Agency. This led to an inaccessibility of appropriate and timely healthcare for several groups including the elderly—and insufficient possibilities to protect/shield for community transmission (Pashakhanlou, 2021). There was no democratic and multi-disciplinary decision-making process nor transparent and accurate communication to the public. In political analogy this problem with *evasive accountability*, *autocratic governance*, *cover-ups* and *secrecy* is referred to as “sovietisation” (Sörensen, 2020). A one-way trust in “the authorities” was expected from the entire population. Yet, the authorities did not trust the people enough to be transparent in their communication, strategy, and outcomes. Nevertheless, all responsibility was with the public using non-binding recommendations—not the authorities. From a legal perspective, the use of *soft law instruments* is also confusing, since “non-binding rules do not offer the traditional formal mechanisms for legal protection, the publication of norms or accountability” (Wenander, 2021). Not including citizens in the decision-making process about the strategy also contributes to a deconstruction or erosion of democracy. Criticism against the Swedish strategy did not follow any clear political ideologies in Sweden, yet the defenders of the Swedish strategy appeared strongly nationalistic (without a clear left- or right-wing orientation).

### Outcomes of the Swedish strategy on a societal level

The Swedish pandemic response included multiple forms of *pandemic prioritisation* (Nielsen, 2021), although not in its expected sense: *Social-welfare prioritisation* seemed to fit more economically advantaged instead of the usual vulnerable and socially disadvantaged. Considering *severity prioritisation*, severe cases have been deprioritised, not receiving adequate healthcare (e.g., ICU), and individuals with comorbidities were less likely to receive optimal care. *Age-based prioritisation* runs against the core-prioritarian idea, and the handling in elderly care was a clear example.

The Swedish strategy included age-based recommendations for voluntary quarantine and isolation (for those 70 years or older on March 16, 2020)—with the aim to protect a vulnerable group (Nilsson et al., 2021). Yet, this has implied deprivation of previously assigned individual responsibility and consequent autonomy—which may contribute to long-term poorer health among these older adults (Nilsson et al., 2021). Therefore, the effectiveness of age as a principle for policy making in the Swedish neo-liberal society has been questioned (Nilsson et al., 2021). It has also been shown that sheltering older individuals without significant social distancing in other age groups is not sufficient to be protective (Roxby and Gure, 2020; Brandén et al., 2020). As mentioned above, the impact of the pandemic on children was also disregarded.

There were also reports of *inequality* and *social injustice* as a consequence of Sweden’s response—especially with elderly, people in nursing homes, individuals with a migration background and socio-economically less-advantaged groups (also of younger age) being affected by excess mortality (Khorram-Manesh et al., 2020; Rostila et al., 2021; Strang et al., 2020; Calderón-Larrañaga et al., 2020; Hansson et al., 2020, 2021e). This inequality narrative was openly communicated by officials including the Public Health Agency, claiming that “The corona infection in the nursing homes may have been spread by staff with poor command of the Swedish language”, “we have larger spread because of the larger immigrant population”, “only the foreigners get ill”, “only people looking like tourists wear face masks in public”.(McKee et al., 2020; Hansson et al., 2020; Tegnell, 2020; Capar, 2020; Höglund, 2020). No significant efforts were taken to decrease these disparities. The clustered spread (in particular in groups with lower socio-economic status or ethnic minority groups (2021e)) did not fit the assumption of a relatively rapid spread as for influenza (or smallpox), which was the underlying pattern of the Public Health Agency pandemic plans—and the apparent Swedish strategy to allow continuous spread without overwhelming healthcare (Lindström, 2020a; McKee et al., 2020; Hansson et al., 2020; Tegnell, 2020; Capar, 2020; Höglund, 2020).

Together with the underlying societal framework supporting this acceptance of “it’s only the foreigners” may have led to an increase in *nationalism* (and even xenophobia?), and maybe contribute to “*Welfare chauvinism*”, the antipathy against the benefits of the welfare system being shared with immigrants and their descendants—as described in a Danish study on the role in the pandemic in the Nordic Welfare states (Larsen and Schaeffer, 2021). In addition, the unequal access to healthcare, the consequent poorer outcomes for certain groups, and its’ general acceptance by the public seems to support *Social Darwinism philosophy* (Alston, 2020), the so-called “survival of the fittest”.

There were never strong feelings of solidarity in the Swedish population, as in “everyone together against the virus” as in other countries especially during the first six months of the global pandemic (Borrud, 2020). The main message seemed that those who are more vulnerable are not going to be protected by the State (since they should take their own measures and isolate). The rest of the population should live their lives relatively uninterrupted. It has been a largely held belief by the Swedish public and perpetuated by the authorities that if people are not symptomatic, they cannot spread COVID-19 (2021h). The Swedish strategy was consequently tailored to accommodate the middle/upper class. Younger and wealthier individuals should be restricted as little as possible in their daily movements while less-advantaged people could not work from home (Nygren and Olofsson, 2020).

### Outcomes of the Swedish strategy on a societal level

On May 21, 2020, the British independent sustainability rating agency *“Standard Ethics”* lowered the ethical rating of Sweden, since the Swedish health policy did not comply with the WHO recommendations (2020p). This produced additional risks for the Swedish and European populations, according to the analysts—and the strategy is not collaborative with the European Union (2020p).

In November 2020, the Organisation for Economic Cooperation and Development’s (*OECD*) and the *European Union* ranked Sweden lowest among 35 European countries considering multiple COVID-19 management metrics including lowering the spread of infection, reducing people’s mobility, and discharging ICU patients (Bjorklund, 2020, 2020k). The OECD states that part of the society is undervalued and under-resourced, referring to the situation in elderly care (2021c).

## Discussion

Sweden is a prosperous and highly developed country, which has invested strongly in healthcare and research over recent decades. Despite the available competence and infrastructure both in academic and industrial settings, these (human) resources were scarcely used during the COVID-19 pandemic. We argue that there was failure of science advice from the start with “COVID-denial” and disregard of scientific evidence (2021e, Miller, 2020). The Government took a passive, hands-off stand, delegating responsibility to the Public Health Agency. The Public Health Agency did not base its advice on scientific evidence but on pre-conceptions on influenza pandemics and herd-immunity—relying primarily on a small advisory group with a narrow disciplinary focus and too limited expertise. A multi-disciplinary, democratic scientific discussion or debate has not taken place, leading to the rise of “shadow science advisory bodies.” None of the official actors took notion of what could have been done better, and no one took responsibility for the results. This institutional rigidity is illustrated by the self-protective reaction to external critique. The Swedish strategy was considered “internationally superior” from the beginning and should not be questioned, a position fuelled by the Swedish mainstream (and state-sponsored) media (Bjurwald et al., 2021; Andersson and Aylott, 2020).

Transparency and accurate information to the public were not a priority—with most communication aimed to “not spread fear” or increase social unrest. If the government and authorities are not honest and transparent towards the public about the virus, how it spreads and the risk to them (individually and collectively in society), then how can individuals make responsible, informed decisions? Protecting the “Swedish image” (*Sverigebilden*) nationally and internationally has appeared to be more important than protecting the lives of Swedish residents, including healthcare workers, elderly, individuals with risk factors (e.g., comorbidities), minority groups and the socio-economically less advantageous. This is evidenced by the high excess mortality in these groups, lack of proper protective personal equipment, and denial of healthcare. There remains a lack of ethical consciousness and the skill to include ethical reasoning in decision-making processes; and lack of compassion for the victims of the pandemic (Bergmann, 2021b; Bergmann, 2021a).

The Swedish strategy was not pro-active in stopping the spread of the virus and this was acknowledged as never being the aim. Authorities reacted slowly and reactively, not dynamically, and never changed paths abruptly. It could be argued that the Swedish strategy was quite efficient and successful if the aim was to let the infection spread at a moderate pace in society. Yet the projected “natural herd-immunity” levels are still nowhere in sight 1.5 years after the start of the pandemic. Herd-immunity does not seem within reach without widespread vaccinations, and with newer variants it may be unlikely. While maintaining healthcare demand at acceptable levels was a stated goal, healthcare resources were under major pressure, with numerous reports on staff shortages, individuals being denied healthcare (in elderly care and outside), and overwhelmed hospitals during 2020 leading to postponed (urgent) healthcare for non-COVID related diseases (2021d, Bark, 2020). Even accurate numbers on COVID-19 infections and deaths were no priority, as clear from the restricted access to (often suboptimal) testing and healthcare, lack of contact-tracing to identify suspected cases, delays in reporting and non-sensitive case-definitions (leading to underestimations).

The cost in terms of infections and deaths of this pandemic in Sweden has been larger in some other more densely populated and more centrally located countries, yet is still markedly higher than in the other Nordic countries (Rizzi et al., 2021; Nanda et al., 2021) and long-term health and societal effects cannot be ignored. Several studies have shown that the human costs would have been significantly lower in Sweden if stricter measures had been implemented, without more detrimental impacts on the economy (Kamerlin and Kasson, 2020; Sjödin et al., 2020; Sheridan et al., 2020; Born et al., 2021b; Amiri, 2021; Born et al., 2021a). The Swedish strategy has not shown to be superior in any measurable aspect compared to the Nordic neighbours or internationally (Balmford et al., 2020, 2020k; Braithwaite et al., 2021; Bjorklund and Ewing, 2020). This Swedish laissez-faire strategy has had a large human cost for the Swedish society. However, relying on public responsibility seemed to have worked to some extent as a consequence of the Swedish high trust in authorities.

The Swedish strategy has also been at the base of the controversial *Great Barrington Declaration* (published October 4, 2020) aiming for natural herd-immunity by letting the infections spread in a “controlled way” in society (Kulldorff et al., 2020), with several of the initiators/defenders having strong ties to Sweden (2021e). This strategy is considered internationally as unscientific, unethical, and unfeasible (Aschwanden, 2020; Aschwanden, 2021; Khalife and VanGennep, 2021; Sridhar and Gurdasani, 2021). Consequently, we argue that the Swedish strategy and several of its supporters have undermined efforts to suppress the infection in other countries (Kulldorff et al., 2020; Mccurry, 2020; Giesecke, 2020; Vogel, 2020, Bjorklund and Ewing, 2020).

The Swedish approach also contributed to an important polarisation in Sweden, between strong supporters and those raising critical questions. In the latter group, there was a disillusion in the authorities and healthcare system, and a broken trust in the *Welfare* State. Yet, a large part of the Swedish society remained highly confident in the authorities and Swedish strategy (Helsingen et al., 2020; Johansson et al., 2021).

This pandemic revealed several structural problems in the Swedish society, on political and judiciary level, in healthcare, official media and bureaucracy—with decentralisation, lack of accountability and independence, and withholding accurate and complete information from the public as recurrent problems at different levels (2021e, Yan et al., 2020). The absence of an independent authority or institute exclusively concerned about national infection control also had major consequences, since decision-making by the few involved actors seemed heavily politicised instead of scientific (2021e).

It should be possible to discuss alternative strategies to pandemic handling, such as a natural herd-immunity strategy. Yet, any scientific discussion must acknowledge high-quality multi-disciplinary (inter-)national evidence, and the effects on all levels of society must be acknowledged and considered in policy and decision-making. Despite the mounting evidence, the Swedish authorities still deny an active herd-immunity strategy—and have clearly misled the public about their intentions, ignoring and discrediting international scientific evidence and spreading misleading information. Handling of any national crisis cannot be dictated by a handful of people with limited expertise and narrow disciplinary focus. Discussion, opposition, critical reflection, and especially clear and honest communication to the stakeholders are crucial, especially since there can be consequences across national borders as we have seen during this pandemic. We argue that the protection of human rights of all citizens should have been considered during COVID-19 policy making in Sweden, and that ethical and moral discussions should not be shunned even during crisis management. Globally, there have been tremendous efforts to collect data throughout the pandemic to guide policy making and improve health outcomes, and we have decades of experience with previous outbreaks and epidemics, also in Sweden. The Swedish COVID-19 policy has been an outlier from the start, in Europe and globally, despite its excellent record in healthcare research and prevention. It is clear this is a consequence of the societal structure and changes over the last decades.

Although structural errors also occurred in other countries, the Swedish strategy seemed characterised by the widespread rejection of scientific evidence, despite the competence and resources available in the country (e.g., researchers in academia and industry, testing infrastructure). COVID-denialism was seen in other countries (including anti-masks) (Miller, 2020), however, this was mainstream in Sweden and supported/driven by the authorities. A select group who clearly lacked the multi-disciplinary expertise required to handle all aspects of a pandemic, took on the role as scientists, health officials and political decision-makers—without any opposition or questioning by the political system. Critical questioning, even by internationally renowned scientists and experts, became risky, even dangerous, in a country where conformism was encouraged by the national media.

The Swedish reputation internationally may have been harmed long-term as a result of its non-conforming actions. Its relationship with neighbouring Nordic countries were put under pressure (Martikainen and Sakki, 2021; Johnson, 2021). Despite initiatives to enhance Nordic collaborations and to prepare together for future pandemics (Nordic Council 2019, Nordic Cooperation 2020), there seemed to have been little collaboration and communication considering the strategy for COVID-19 in the Nordic countries, with Sweden taking a clearly different path (Vilhelmsson and Mulinari, 2020). The same occurred in 2009, with the swine flu (H1N1), when Sweden opted for mass-vaccination, and Denmark for vaccinating risk groups (Vilhelmsson and Mulinari, 2020). At that time, Anders Tegnell was working at the National Board of Health and Welfare and one of the main drivers/decision-makers behind the mass-vaccination (together with Johan Giesecke) and was consequently criticised because of the significant number of narcolepsy cases occurring post-vaccination (TT, 2020b).

The repetitive statements strongly claiming that all other countries were wrong or experimenting during the current pandemic, also led to some international tensions (Mccurry, 2020, 2020c), and Sweden’s self-proclaimed position as moral superpower and Life Science Nation could be questioned (Lanz, 2021; Steinfeld, 2021), in particular by its non-cooperative stand and resistance to EU’s corona bonds, ECDC and WHO recommendations (Vogel, 2020, 2021e). The lack of accountability reveals a structural political pathology where no one really can be held accountable for the failures and the loss of all too many lives.

## Conclusion

The Swedish response to this pandemic was unique and characterised by a morally, ethically, and scientifically questionable laissez-faire approach, a consequence of structural problems in the society. There was more emphasis on the protection of the “Swedish image” than on saving and protecting lives or on an evidence-based approach. A strategy was never discussed among all relevant parties, and never implemented nor communicated to the public. In addition, there was an unwillingness and incapacity to admit any failures at all governmental levels; or to take any responsibility for the clearly detrimental outcomes for Swedish society. There were even attempts to revise history by changing, or deleting official documents, communication, and websites, and gaslighting the public. The Swedish authorities involved were not self-critical and did not engage in any official and open dialogue and misled the public by withholding correct information and even spreading misleading information. A small group of so-called experts with a narrow disciplinary focus received a disproportionate and unquestioned amount of power in the discussion, nationally and internationally. There was no intellectual/scientific discussion between stakeholders (including independent experts from different disciplines), and the international advice of WHO, ECDC and the scientific community was ignored and/or discredited.

## Supplementary information

Supplementary material

## Data Availability

All material is available in the manuscript and supplements. The cited emails can be retrieved from the authors upon request.
