# Correction: Evaluation of science advice during the COVID-19 pandemic in Sweden

**DOI:** 10.1057/s41599-022-01254-w

**Published:** 2022-07-15

**Authors:** Nele Brusselaers, David Steadson, Kelly Bjorklund, Sofia Breland, Jens Stilhoff Sörensen, Andrew Ewing, Sigurd Bergmann, Gunnar Steineck

**Affiliations:** 1 Centre for Translational Microbiome Research, Karolinska Institutet, Stockholm, Sweden; 2 Global Health Institute, Antwerp University, Antwerp, Belgium; 3 Department of Head and Skin, Ghent University, Ghent, Belgium; 4 Science Forum COVID-19, Umeå, Sweden; 5 VanaTech Behavioural Science, Älvkarleby, Sweden; 6 Freelance Journalist, Stockholm, Sweden; 7 Freelance Journalist, Washington, DC USA; 8 Oskarström Primary Care, Halmstad, Sweden; 9 School of Global Studies, Gothenburg University, Gothenburg, Sweden; 10 Department of Chemistry and Molecular Biology, Gothenburg University, Gothenburg, Sweden; 11 Department of Philosophy and Religious Studies, Norwegian University of Science and Technology, Trondheim, Norway; 12 Clinical Cancer Epidemiology, Department of Oncology, Gothenburg University, Gothenburg, Sweden

**Keywords:** Health humanities, Science, technology and society, Social policy

Correction to: *Humanities and Social Sciences Communications* 10.1057/s41599-022-01097-5, published online 22 March 2022.

A change has been made to the Abstract to correct a statement about the merger of the Swedish Public Health Agency with the Institute for Infectious Disease Control.

The original text read:

In 2014, the Public Health Agency merged with the Institute for Infectious Disease Control; the first decision by its new head (Johan Carlson) was to dismiss and move the authority’s six professors to Karolinska Institute. With this setup, the authority lacked expertise and could disregard scientific facts.

The corrected text now reads:

In 2014, the Public Health Agency, after 5 years of rearrangement, merged with the Institute for Infectious Disease Control, with six professors leaving between 2010 and 2012 going to the Karolinska Institute. With this setup, the authority lost scientific expertise.

