# Supplementary material for: Evaluation of science advice during the COVID-19 pandemic in Sweden
Source: Humanit Soc Sci Commun. 2022 Mar 22;9(1):91. doi: 10.1057/s41599-022-01097-5 (PMC11592722; doi:10.1057/s41599-022-01097-5)
Supplement: Supplementary file 1 — Supplementary material [file 41599_2022_1097_MOESM1_ESM.docx]

**Evaluation of Science Advice in the COVID-19 pandemic in Sweden: Supplementary material**

**Authors:** Nele BRUSSELAERS,^1,2,3,4^, David STEADSON,^5^ Kelly BJORKLUND,^6^ Sofia BRELAND,^7^ Jens STILHOFF SÖRENSEN,^4,8^ Andrew EWING,^4,9^ Sigurd BERGMANN,^4,10^ Gunnar STEINECK,^4,11^

**Affiliations**

1. Centre for Translational Microbiome Research, Karolinska Institutet, Stockholm, Sweden
2. Global Health Institute, Antwerp University, Antwerp, Belgium
3. Department of Head and Skin, Ghent University, Ghent, Belgium
4. Science Forum COVID-19, Sweden
5. VanaTech Behavioural Science, Älvkarleby, Sweden
6. Freelance Journalist, Sweden and United States
7. Oskarström Primary Care, Halmstad, Sweden
8. School of Global Studies, Gothenburg University, Gothenburg, Sweden
9. Department of Chemistry & Molecular Biology, Gothenburg University, Gothenburg, Sweden
10. Department of Philosophy and Religious Studies, Norwegian University of Science and Technology, Trondheim, Norway
11. Clinical Cancer Epidemiology, Department of Oncology, Gothenburg University, Gothenburg, Sweden

**Correspondence**

Nele Brusselaers, Centre for Translational Microbiome Research, Biomedicum 8A, Solnavagen 9, 17165 Stockholm, Sweden. (nele.brusselaers@ki.se)

# **Supplementary material**

## **Supplement 1: Study setting – Sweden**

**Supplement 2: Supplementary methods**

## **Supplement 3: Most prominent official actors in the Swedish crisis management and pandemic handling**

## **Supplement 4: Global, European, and Swedish timelines**

## **Supplement 5: Testing and contact-tracing in Sweden**

## **Supplement 6: Consequences of the Swedish approach in health- and elderly care.**

## **Supplement 7: Children and schools**

## **Supplement 8: Scientific Advisory Groups during the pandemic**

**Supplement 9: Systemic violation of the rule of law and human rights**

## **Supplement 1: Study setting – Sweden**

The Nordic region includes Sweden, and its three neighbours, Denmark, Finland, and Norway, as well as Iceland and three autonomous territories belonging to Denmark (Faroe Islands and Greenland); and Finland (Åland). With approximately 450,000 square kilometres (173,860 sq. mi), Sweden is the largest country in Northern Europe and the third-largest country in the European Union (EU). Sweden is ranked 7^th^ based on the 2020 Human Development Index (HDI) with a HDI value of 0.945 (values between 0.800-1.000 are categorized as “very high” human development). Sweden has a population of somewhat more than 10 million inhabitants and has a low population density of 23 inhabitants/km^2^ (59/mi^2^). The majority of the population lives in three larger metropolitan regions (Stockholm, Gothenburg, Malmö) while population density is sparse in many places.(2019d) Consequently, population densities are markedly higher after calibration for areas and habitation patterns. There are 21 counties and 290 municipalities in Sweden; and the proportion of single-member households is large.(2019d)

*Swedish healthcare*

The Swedish share of the Gross Domestic Product (GDP) allocated to health spending (excluding capital expenditures) was 11% in 2019, while the OECD average was 9%.(2021z) Private health insurance plays a “negligible” role in Sweden according to a 2015 OECD report.(2015a). Yet, a growing proportion (approximately 6-7% in 2020) has additional private health insurance with faster access to health care such as shorter waiting times for some surgical procedures.(2021z, 2021k) Over the last 30 years, Sweden has increasingly privatized tax-financed public services (for-profit enterprises), in particular in elderly care.(Diderichsen, 2021, Granberg et al., 2021) Previously, the Swedish so-called “welfare state” was characterized by tax-financed and equally distributed healthcare – yet reforms led to a “highly decentralized, deregulated and liberalized welfare state”, with a significant part played by private actors.(Granberg et al., 2021) The decentralization of healthcare is regulated by the Health and Medical Service Act (*Hälso- och sjukvårdslag* (1982:763), last updated in 2017).(2021z) The 21 Swedish county regions exercise a degree of self-governance, and they are in turn composed of a number of municipalities.(2021z) The National Board of Health and Welfare (*Socialstyrelsen*) is the Government agency under the Ministry of Health and Social Affairs responsible for compiling information and developing standards to ensure good health, social welfare and high-quality health and social care for the whole population.(2021z) Swedish law states that every regional council (political bodies with elected representatives) must provide residents with good-quality medical care, and work to promote good health for the entire population.(2021z) The municipalities are also responsible for care of the elderly and care for people with physical disabilities and psychological disorders, and they are to provide support and services after discharge from hospital, and school healthcare.(2021z)

Sweden has 86 hospitals, including seven university hospitals.(2021a) Overall, 204 hospital beds for “curative care” were available per 100,000 inhabitants in Sweden (2017);(2019b) and 5.8/100,000 intensive care unit (ICU) beds (2014), compared to the European average of respectively 372 and 11.5/100,000 inhabitants.(Rhodes et al., 2012, 2018b) The Swedish amount of hospital beds per capita was the lowest in Europe, and the second lowest for number of ICU beds, and has decreased drastically in the recent decades (in 2014 still 521/1000,000 hospital beds).(2018b, 2019b, 2019a)

The decentralized approach of Swedish healthcare means several aspects of public health organization and responsibilities are delegated to the regional and municipal levels.(Aluttis et al., 2013) The 21 regions have different politicians governing health care, and there are 21 separate patient registries. Sweden has several national state agencies (see **Supplement 3**), in some cases with outreach to the regional level, 21 County Councils/Regions on the regional level and 290 municipalities on the local level. Budgets for public health are located at different levels and there are no systematic accounts summing up the total funding.(Aluttis et al., 2013) This is partly due to the absence of a clear and generally agreed definition of what to include as ‘public health’.(Aluttis et al., 2013) Sweden adopted a national public health policy in 2003 (updated in 2008), which states that public authorities are guided by 11 objectives covering the most important determinants of Swedish Health, including good living conditions, health-promoting living environments and living habits.(Aluttis et al., 2013, Linell et al., 2013) In 2008, the core content remained unchanged, yet individual choice and responsibility were added.(Aluttis et al., 2013) The additions to this policy focused on children, young people and the elderly.(Aluttis et al., 2013)

*Sociocultural, economic, and political structures and ideologies in Sweden*

According to the World Values Survey (WVS), Sweden has been globally one of the most ***non-traditional***, ***secularized*** (non-religious), ***rational***, ***post-materialist*** and ***post-modern*** countries in the world during the past decades.(Inglehart, 2018, Lindström, 2020, 2020u). It is also the country with the highest value for self-expression (in contrast to survival values).(2020u, Inglehart, 2018, Lindström, 2020). *Post-materialism* refers to the larger concern about individual freedom and human rights, arising in the 1960s-1970s in many Western societies in contrast to materialist values dominating in times when material and physical security were scarce.(Lindström, 2020) *Post-modern* cultures are a reaction to *modern* cultures.(Lindström, 2020) In post-modern cultures, authority, rationality, science and engineering are considered typical for the West and Western materialistic culture.(Lindström, 2020) With *post-modernism*, new values and lifestyles are embraced, with increased tolerance of ethnic and cultural diversity, and sexual and individual choices.(Lindström, 2020) Considering science, the *postmodern* view also emphasizes subjective personal feelings of “what is true” rather than the *modern* belief that there is only one objective truth.(Lindström, 2020, Lindström, 2021, Orlowski and Goldsmith, 2020)

Sweden also ranks globally among the countries with the lowest regard to the elderly based on pre-pandemic surveys.(2020u, Larsson and Hartig, 2015) ***Ageism***, defined as prejudices or stereotypical perceptions based on a person's age and which can lead to discrimination, is a bigger problem in Sweden than in the other Nordic countries.(Larsson and Hartig, 2015) When Swedish people are asked to rank the social position of different age groups in their country, the position of the elderly (70+ years) is ranked as the second lowest in the world. The elderly are also considered to have a much weaker position in society than young adults.(Larsson and Hartig, 2015) This may be a consequence of prevailing individualism and rapid digitalisation and technological development, and weaker ties between the different generations (the family is not at the centre as in several other countries).(Larsson and Hartig, 2015) In the Swedish society, aging is mainly regarded as a biological process of decay or decline, dehumanising the senior in the eyes of the rest of society, leading to marginalization and discrimination; considering the senior as a passive object.(Larsson and Hartig, 2015)

Sweden has been described as a ***liberal*** or increasingly ***neoliberal democracy***, which refers to economic liberalism and free-market capitalism – with policies aimed at economic liberalization (including privatization, deregulation, globalization, free trade) and reduced Government spending to increase the role of the private sector in society and the economy – also characterized by “just in time” deliveries (on a global market).(Granberg et al., 2021, Nygren and Olofsson, 2021, Sörensen, 2020, Etzioni, 2021, Farina and Lavazza, 2020, Gerle, 2021, Korhonen and Granberg, 2020)

Although Sweden has developed a strong civil society with elements of ***participatory democracy*** and values of ***equality*** and ***liberalism***, the country at the same time practiced a strong ***top-down consensus-culture***, with a clear avoidance of conflicts, on a political, mass media, cultural and corporate level, even described as balancing on the border of totalitarianism, or as a totalitarian democracy.(Lindström, 2021, Sörensen, 2020, Rosenberg, 2002) The long history of consensus culture allowed the participation of citizens but nurtured at the same time a lack of managing conflicts and coping with otherness.(Rosenberg, 2002) The increase in ***autocratic elements*** may also undermine Sweden’s traditional democracy.(Lindström, 2021, Bergmann, 2020, Rosenberg, 2002) The modern Sweden offers a combination of ***liberal individualism*** on the one hand and ***cultural conformism*** on the other, where the state in the field of popular health has educated its’ citizens strictly considering food, dental care, infant nutrition and much more.(Lindström, 2021, Bergmann, 2020) Individual freedom is deeply connected to ***communitarian*** ***conformism*** and ***conventionalism***.(Lindström, 2021, Bergmann, 2020)

The ***Swedish leadership*** is more informal and consensual than the Anglo-American leadership style, with less focus on directive leadership and more on group decisions.(Appelgren, 2021, Petridou and Zahariadis, 2020) Swedish leadership is more about involvement, and although time-consuming, it should lead to rapid acceptance when decisions are made.(Appelgren, 2021) Honesty and trustworthiness are appreciated in Swedish leaders, and they should work for the “common good”, be team-oriented, and pro-collaboration.(Appelgren, 2021) A typical Swedish leader is not supposed to be self-centred, status-conscious or non-participative.(Appelgren, 2021) However, ***political parties*** are heavily top-down governed with very little consensus or involvement – described as authoritarian party pyramids.(Pålsson, 2011, Appelgren, 2021, Petridou and Zahariadis, 2020)

Because Sweden is a ***highly export-dependent*** country, Swedish foreign policy has aimed to promote Sweden as a model since the 1980s,(Sörensen, 2020) with a special Government agency (the Swedish Institute) to promote the “Swedish Image” (***Sverigebilden***) internationally – Sweden as a moral and humanitarian superpower.(Sörensen, 2020)

In addition, the Swedish population has uniquely high levels of ***social and institutional trust***.(Esaiasson et al., 2020, Etzioni, 2021, Farina and Lavazza, 2020, Bylund and Packard, 2021, Hayry, 2021, Josefsson, 2021, Ludvigsson, 2020b, Nanda et al., 2021, Nielsen and Lindvall, 2021, Pierre, 2020, Woelfert and Kunst, 2020, Rothstein, 2005 (online 2009), Trägårdh, 2013). The perspective of ***Swedish exceptionalism*** in relation to the rest of the world has also been strong in Swedish society since the 1970s,(Lindström, 2020) suggesting a covert or hidden ***nationalism*** – especially considering Swedish “superior” rationality, secularism, tolerance, flexibility and openness.(Nygren and Olofsson, 2021, Sörensen, 2020, Granberg et al., 2021)

*Sweden as “Welfare State” and “leading Life Science nation”*

The Nordic health system is modelled on a framework including public participation and patient choice.(Biddle et al., 2021) The Nordic societies are “varieties of a common ***neo-corporatist model’’*** with a strong and active state closely interacting with civil society organizations; highly coordinated labour market policies and a “generous welfare state”.(Biddle et al., 2021)

Sweden has regularly been praised as one of the “Nordic Welfare States”, with a universalistic and all-encompassing approach to ***welfare*** and ***egalitarianism***.(Greve et al., 2020, Lindblad et al., 2021) Sweden has even been described as an exceptional Welfare State, as one of the most advanced nations in terms of welfare service delivery and quality of welfare production and organization.(Granberg et al., 2021) These Nordic welfare states have been described as ***social democratic*** (comprehensive and generous welfare transfers and social services including child- and elderly care, free public education); promoting economic and gender equality; and with an active labour market policy to enhance labour market participation and re-entering after unemployment.(Greve et al., 2020, Granberg et al., 2021) Although criticized in the 1980s, this welfare state model is considered a “high spenders” model redistributing market incomes considerably through tax and fiscal policies (e.g. generous social benefits including pensions, parental benefits).(Greve et al., 2020) ***Decentralisation***, with more responsibility to the regions and municipalities, however included marketization of key parts of the welfare state, which increased the importance of ***private actors*** in the provision of public services, resulting in an important impact on the ethos of public sector organizations – also described in 2016 as a Swedish “decline in welfare exceptionalism”.(Granberg et al., 2021) The resulting ***highly deregulated*** and ***liberalised welfare*** ***state*** based on a supply-and demand system, just-in-time supplies (including staff), and a strong focus on economic efficiency resulted in far-reaching cost-cutting in the public sector.(Granberg et al., 2021) The municipalities function by local self-Government, also according to the Swedish constitution.(Wenander, 2021) This means infrastructure, housing, business development, schools, public-health education and care for the elderly are heavily decentralized. Although healthcare is a national responsibility, health decisions and implementation are carried out at the regional and municipal level.)(Mens et al., 2021) This has led to a ***fragmented public sector***, with many policy fields dominated by a ***client–contractor model***, a ***mixed private-public management structure***, and citizens as individual consumers of services - yet with an increased focus on freedom of choice.(Granberg et al., 2021)

Sweden also wants to transform towards a sustainable and resilient society, in agreement with the “Global Goals and 2030 Agenda for Sustainable Development” and seek to realize, among others, the human rights of all and achieve gender equality, while balancing the three dimensions of sustainable development: economy, environment and society.(2018a) The third Global Goal on Good Health and Well-being is to “ensure healthy lives and promote well-being at all ages.(2018a)

In addition, the Government describes Sweden also as a “knowledge nation that has long invested in equitable health care, research and innovation” and wants to promote its “national life sciences strategy”. The Government also aims to be a leading life sciences nation, “to improve health and quality of life of the population, ensuring economic prosperity, advancing as a leading knowledge nation and achieving the 2030 Agenda for Sustainable Development”.(2021e) This strategy stresses the need for a greater focus on preventing ill health and disease, and states that the Government is firmly committed to continue to develop “world-class research, research infrastructure, and a healthcare care system of high international standard, competitive industry and world leading innovation.”(2021e)

*Swedish legal system, including infection control regulations*

From a macro-comparative level, the legal systems in the Nordic countries are sometimes described as the “***Nordic legal family*** (as part of the Germanic or Euro-American legal sphere if grouped on a higher level).(Wenander, 2021, Lando, 2001, Husa, 2011) These divisions are based on e.g. common history, ideology, legal style, and argumentation or the “legal mentality” so there are also differences between the Nordic countries particularly considering legal rules.(Husa, 2011) These “Nordic legal systems” are based on a high level of trust in the “wisdom and reasonableness” of the authorities, following a notion of “the good state”.(Wenander, 2021) They are characterized by a collectivist culture, where public authorities can rely on citizens adhering to the letter, but also to the spirit of given rules in good faith.(Wenander, 2021) Compared to other Euro-American legal systems, the legal-formal and doctrinal position of the Nordic legal system is relatively weak with an inherent suspicion towards judge-made rules.(Husa, 2011) Specifically for the Swedish and Finnish legal-cultures, moral questions should be left to the Parliament, not the courts of law – consequently giving more power to politicians than the judiciary.(Husa, 2011) In the Nordic Countries, key constitutional documents or “Constitutional Acts” are written by key governmental institutions even though they are supplemented in various ways by formal amendments, constitutional conventions or other customary rules and praxis – and these are considered at the top of the national hierarchy of legal norms.(Husa, 2011) Yet, only Sweden has many formal constitutional documents, all with *de jure* constitutional status – although some are more important than others.(Husa, 2011) Sweden and Finland also do not recognize the clear principle of separation of powers.(Husa, 2011)

The ***Swedish Constitution*** consists of four fundamental laws: the Instrument of Government, the Act of Succession, the Freedom of the Press Act, and the Fundamental Law on Freedom of Expression.(Wenander, 2021, Nordberg and Mattsson, 2020) The Instrument of Government regulates the state institutions, protection of human rights, forms of legislation and constitutional control – relying on ***undivided popular sovereignty*** and not on a separation of powers.(Wenander, 2021) The role of state powers (constitutional conceptualism) is therefore not clearly described.(Wenander, 2021) The Council of Europe’s Convention on Human Rights is also fully incorporated in the Swedish national law.

A Swedish 'public agency' is governed in 3 ways: 1) The laws including the constitution (*Regeringsformen*, *Yttrandefrihetsgrundlagen*), with its regulations on e.g. recruitment and other laws such as Administrative Law (*Förvaltningslagen*), and specific laws for particular sectors. 2) An "instruction" from the government (*Myndighetens instruction, styrdokument*). 3). The annual "Regulation Letter" (*Regleringsbrev*), which states e.g. priorities and comes with the budget. Both the “instruction” and “regulation letter” are “control documents” (*styrdokument).*

In Sweden, the ***'Freedom of Information laws***’ (*offentlighetsprincip*), under the Freedom of the Press Act, are a cornerstone of the legal system. It means that the public (including individuals and representatives of the media) have the right to access information about the activities of the State, the 21 regions and the 290 municipalities.(2020k) "To encourage the free exchange of opinion and availability of comprehensive information, every Swedish citizen shall be entitled to have free access to official documents." (Chapter 2, Article 1, Freedom of the Press Act)(2015c) This freedom of information through public access of official documents implies that all documents (including emails, meeting agendas, final meeting decisions) are public, yet the right to access may be restricted for confidentiality reasons.(2015b, 2020k)

The ***Communicable Diseases Act*** (*Smittskydslagen*) (2004:168) and the ***Communicable Diseases Ordinance*** (2004:255) include several notifiable diseases (*anmälningspliktiga sjukdomar*) in Sweden – which are divided into different categories, depending on the requirement for mandatory contact tracing, the danger to public health, and the danger to society (*allmänfarliga- or samhällsfarliga*). (2021v, 2021w, 2021t, 2021u) (2021v, 2021w, 2021t, 2021u)(2021v, 2021w, 2021t, 2021u) According to the Communicable Diseases Act, a physician or laboratory, that suspects or establishes a case of a notifiable disease, must promptly report this to the regional infection control physician (*smittskyddsläkaren*) and the Public Health Agency.(2021w, 2021l) According to this Act (kap. 2, 7 §) the Government or the Agency they decide upon, can write injunctions to exempt or limit/restrict the obligation to report cases of notifiable diseases.(2021w, 2021l) The Public Health Agency has the official responsibility to maintain and coordinate the infection control on a national level. For notifiable diseases which are considered a danger to the public *(samhällsfarliga*), testing and contact tracing must be coordinated by the regional infection control physician (*smittskyddsläkaren*), according to these regulations – which also state that geographical areas can be placed under quarantine by the Public Health Agency if required to stop the spread of the disease.(2021l)

**Supplement 2: Supplementary methods**

The systematic review was conducted to identify all relevant published peer-reviewed scientific papers (not restricted to types of studies, including original studies, letters, opinion pieces) describing the COVID-19 pandemic situation in Sweden during 2020. To not miss relevant articles which may compare multiple countries, the search itself was expanded to the Nordic region.

Official government documents were screened and manually searched for specific information.

Because of the important role of the mainstream media in the COVID-19 pandemic in Sweden and internationally, we also identified relevant newspaper articles and other media publications – especially to have relevant references for transparency purposes. If we could find an international reliable source, this was preferred – especially if it was in English. The following newspapers are the main four newspapers in Sweden: Dagens Nyheter (DN), Svenska Dagbladet, Aftonbladet and Expressen.(2021h) Svenska Radio and SVT (Sveriges Television/Swedish Television) are the official national radio and television stations.

The systematic search was limited to the period of January 2020 to July 8, 2021 since the aim was to focus on the first year of the pandemic (until end December 2020). Only articles in English or Swedish were considered eligible. This resulted in 2771 articles listed in PubMed and 1167 articles listed in the Web of Science.

Citation tracking was performed for relevant papers, and additional specific author searches were conducted for key players in the Swedish COVID-19 debate. Scientific papers retrieved through other sources were also considered if relevant.

**Search string PubMed**:

(Swede*[tiab] OR Swedish[tiab] OR Nordic[tiab] OR Iceland*[tiab] OR Scandinavia*[tiab] OR Denmark[tiab] OR Danish[tiab] OR Finland[tiab] OR Finnish[tiab] OR Norwegian[tiab] OR Norway[tiab] Sweden [Affiliation] OR Denmark [Affiliation] OR Finland [Affiliation] OR Norway [Affiliation] OR Iceland [Affiliation])

AND (covid[tiab] OR corona[tiab] OR SARS-CoV-2[tiab])

**Search string Web of Science:**

(Sweden OR Swedish OR Nordic OR Iceland OR Icelandic OR Scandinavia OR Scandinavian OR Denmark OR Danish OR Finland OR Finnish OR Norwegian OR Norway) AND (covid OR corona OR SARS-CoV-2)

## **Supplement 3: Most prominent official actors in the Swedish crisis management and pandemic handling**

Ten national authorities were identified as major players in the policy making and implementation of the COVID-19 pandemic management during 2020, with their official descriptions as noted on their respective English websites (last accessed July-August 2021).

*Swedish Government (Regeringen), Government Offices (Regeringskansliet) and Parliament (Riksdag)(2021t, 2021o)*

Sweden is officially a ***democratic constitutional monarchy***, with the King (Carl XVI Gustav, °1946, ascended the throne in 1973) being head of state. He has several responsibilities, e.g., during the opening of the parliament each autumn. He also heads a meeting in the Committee of Foreign Affairs each Thursday. Yet, important executive power has been transferred from the head of state to the Prime Minister and Government, and the King may not be involved in statements on political issues since he only has a formal role.

The political system (Government and Parliament) is based on an elected multi-party system, and a ***parliamentary democracy***, stating all public power proceeds from the people. At the national level, the people are represented by the Parliament which has legislative power. The Government implements the Parliament’s decisions; and draws up proposals for new laws or law amendments. The Parliament has 349 members who are elected every four years, and with eight political parties represented during the 2018-2022 electoral term.

The government agency “the ***Government Offices”*** acts as the Government's staff and supports the Government in governing Sweden and implementing its policies.

The Government Offices include the Prime Minister's Office (*Statsrådsberedningen)*, the 11 ministries (*departementen*) and the Office for Administrative Affairs (*Förvaltningsavdelningen)*. The Government Offices have approximately 4 500 employees, some 200 of whom are political appointees. When there is a change of government, the political appointees resign while the non-politically recruited officials retain their positions.

The Prime Minister's Office, headed by the Prime Minister, leads and coordinates work in the Government Offices and is responsible for coordinating Swedish EU policy. Former union boss, Stefan Löfven (Social Democrats), was elected Prime Minister in 2014. After the 2018 elections, Löfven formed a minority Government with the Green Party and relied on support and confidence from the Centre Party, Liberals, and the Left Party, known as the January Agreement. On August 22, 2021 Löfven announced he will resign as party leader and Prime Minister in November 2021.(Thebault, 2021, Henley, 2021)

*Government agencies (myndigheter)*

A “myndighet” (“authority/agency”) is an institution that is part of the public administration or the administration of justice (national level). Each ministry is responsible for a number of government agencies tasked with applying the laws and carrying out the activities decided by the Parliament and the Government.

Every year the Government issues appropriation directions for the government agencies. These set out the objectives of the agencies' activities and their available budget. The Government therefore has quite substantial influence on directing the activities of government agencies, but it has no powers to interfere with how an agency applies the law or decides in a specific case. The government agencies take these decisions independently and report to the ministries. In many other countries, a minister has the power to intervene directly in an agency's day-to-day operations. This possibility does not exist in Sweden, as 'ministerial rule' is prohibited.

The Government is responsible for recruiting and appointing a Director General for each government agency.

*The Ministries (departement) and a selection of their government agencies (myndigheter) – with focus on the most relevant ones during the pandemic*

1. Ministry of Culture
2. Ministry of Defence
3. Ministry of Education and Research

- The **Swedish National Agency for Education** (*Statens skolverk*). (2021aa) (2021aa) (2021aa) (2021aa) (2021aa) (2021aa) (2021aa) (2021aa) (2021aa) (2021aa) (2021z) (2021z) (2021z) (2021z) (2021z) (2021z) (2021y) (2021y) (2021y) (2021y) (2021y) (2021y) The National Agency for Education is the central administrative authority for the public school system, publicly organized preschools, school-age childcare and for adult education. The agency is headed by Director General Peter Fredriksson*.*
- The Swedish Research Council (*Vetenskapsrådet*)

1. Ministry of Employment

- Ombudsman for Children (*Barnombudsmannen, BO*)
- Swedish Work Environment Authority (*Arbetesmiljöverket*) (2021ab) (2021ab) (2021ab) (2021ab) (2021ab) (2021ab) (2021ab) (2021ab) (2021ab) (2021ab) (2021aa) (2021aa) (2021aa) (2021aa) (2021aa) (2021aa) (2021z) (2021z) (2021z) (2021z) (2021z) (2021z) The Swedish Work Environment Authority is a regulatory authority with the mandate from the Government and the Parliament to ensure that companies and organizations abide laws regarding work environment and working hours. The agency is headed by Director General Erna Zelmin-Ekenhem.

1. Ministry of Enterprise and Innovation
2. Ministry of Environment
3. Ministry of Finance

- **Statistics Sweden** (*Statistiska centralbyrån*): (2021r) (2021r) (2021r) (2021r) (2021r) (2021r) (2021r) (2021r) (2021r) (2021r) (2021q) (2021q) (2021q) (2021q) (2021q) (2021q) (2021p) (2021p) (2021p) (2021p) (2021p) (2021p) (2021o) Its main task is to supply users and customers with statistics for decision making, debate and research. This agency procures and distributes the statistics and is responsible for coordinating the system for official statistics and other government statistics in Sweden. The agency is headed by Director General Joakim Stymne.
- County administrative boards (*Länsstyrelserna*)

1. Ministry of Foreign Affairs

- **Swedish Institute** (*Svenska Institutet*): (2021y) (2021y) (2021y) (2021y) (2021y) (2021y) (2021y) (2021y) (2021y) (2021y) (2021x) (2021x) (2021x) (2021x) (2021x) (2021x) (2021w) (2021w) (2021w) (2021w) (2021w) (2021w) This institute promotes interest and trust in Sweden around the world and its core activities involve analysing how foreign target groups perceive Sweden, and how this affects opportunities for Swedish actors abroad. It provides expert support to both private and public actors wishing to communicate the image of Sweden (“***Sverigebilden***”) and Swedish skills. “Sverigebilden” should be characterized by innovation, creativity, and sustainability. The agency is headed by Director General Madeleine Sjöstedt.

1. Ministry of Health and Social Affairs

- **Public Health Agency** (*Folkhälsomyndigheten*)*(2021m)* See below.
- **National Board of Health and Welfare** (*Socialstyrelsen*): (2021q) (2021q) (2021q) (2021q) (2021q) (2021q) (2021q) (2021q) (2021q) (2021q) (2021p) (2021p) (2021p) (2021p) (2021p) (2021p) (2021o) (2021o) (2021o) (2021o) (2021o) (2021o) (2021n) (2021n) This agency has a wide range of duties within the fields of social services, health and medical services, patient safety and epidemiology. The agency also, among other things, issues licenses for medical personnel. The agency is headed by Director General Olivia Wigzell.
- Medical Products Agency (*Läkemedelsverket*)
- **Health and Social Care Inspectorate** (*Inspektionen för vård och omsorg, IVO*): (2021f) (2021f) (2021f) (2021f) (2021f) (2021f) (2021f) (2021f) (2021f) (2021f) (2021f) (2021f) (2021f) (2021f) (2021f) (2021f) (2021f) (2021f) (2021f) (2021f) (2021f) (2021f) This inspectorate is responsible for supervising health care, social services and activities under the Act concerning Support and Service for Persons with Certain Functional Impairments (*LSS*). *IVO* is also responsible for issuing certain permits in these areas as well. Its supervision covers the processing of complaints concerning, for example, the reporting of irregularities in health care and social care (called lex Maria and lex Sarah reports, respectively) and the municipal obligation to report non-enforced decisions. The agency is headed by Director-General Sofia Wallström.
- Swedish Social Insurance Agency (Försäkringskassan)

1. Ministry of Infrastructure
2. Ministry of Justice

- Swedish Security Service
- Swedish courts
- **Swedish Civil Contingencies Agency** (*Myndigheten för samhällsskydd och beredskap, MSB*). This agency is responsible for issues concerning civil protection, public safety, emergency management and civil defence. It includes helping society prepare for major accidents, crises, and the consequences of war. The agency is headed by Deputy Director General Camilla Asp. Dan Eliasson resigned as Director General on January 6, 2021 after it had been made public that he had vacationed abroad several times during the ongoing pandemic.(2021i)
- The Swedish Police
- Chancellor of Justice
- Starting on January 1, 2022: **Agency for Psychological Defence** (the official name in English is not available yet). This agency will be responsible for coordinating, developing, and supporting different agencies and other actors’ operations within Sweden’s psychological defence. These responsibilities used to belong under MSB.(2021j) The new agency should also contribute directly to strengthening the population’s resilience in terms of psychological defence. The agency is responsible for these tasks during peace, at heightened preparedness and ultimately in war.(2021n) The prime-minster (Löfven) stated in 2018 that “The starting point is to preserve our open society's free exchange of knowledge and information. That is a precondition for our democracy and rule of law.”(2018c)

*The Government’s Crisis management*

The Government’s Crisis Management involves the relevant ministry (depending on the nature of the crisis), and the State Secretaries (*statssekreterare*) who are the administrative heads of the ministries. These State Secretaries are the highest public officials in each ministry under the minister. This Crisis Management system was implemented after the 2004 Tsunami, because of a very critical report from the Disaster Committee (*Katastrofkommissionen or Hirschfeldt-commission*) criticizing the lack of coordination between the Ministries, and passivity of all Ministries not actively looking for information - a similar situation to after the 1994 disaster when a ferry between Sweden and Estonia sank.(2005) This Disaster Committee began in 2005, with hearings in Constitution Committee (*Konstitutionsutskottets*) in 2005-6.(2007) In 2007 it was proposed to integrate the new crisis function in the Government Offices (*Regeringskansliet),* i.e. all the ministries. Each Ministry was supposed to have responsibility within their area, with coordination by the Prime Minister’s office (*Statsrådsberedningen*).(2007) It was decided that the Prime Minister must have a special office assisting him and specializing in crisis management, i.e. the Office of Crisis Management (*Kansliet för Krishantering*).(2007) This was installed in 2008 to both coordinate and help the various Ministries in their work, including seeking and coordinating information. The responsibility was moved in 2014 to the Minister of Interior (*Inrikesministern*) located at the Ministry of Justice, and the Chief Civil Servant (*Chefstjänsteman*) is responsible. S/he is directly under the State Secretary in the Ministry, who in turn is directly under the Minister of Interior (in the Ministry of Justice). The Strategic Communication Group (*Gruppen för Strategisk Samordning or GSS*) is a group of state secretaries (from the relevant Ministries in the crisis) and headed by the State Secretary of the Minister of Interior (in the Ministry of Justice). The Office of Crisis Management serves the GSS, and never worked as a separate staff or unit. In 2008 the Crisis Management Council (*Krishanteringsråd*) was also established, again led by the State Secretary under the Minister of Interior. It also consists of the Chief of the Police, The Supreme Commander, and some representatives for some other agencies (including Socialstyrelsen, The Swedish Civil Contingencies Agency (Myndigheten för samhällsskydd och beredskap [MSB], and Svenska Kraftnät, i.e., the authority responsible for ensuring that the Swedish power system is sustainable, safe, and cost-effective.). This Crisis Management Council has bi-annual meetings. Apart from this each Ministry shall have its Crisis Management Group including an operations cell (i.e., a civil servant on standby/duty call).

*The Public Health Agency of Sweden (Folkhälsomyndigheten [FoHM])(2021m)*

The Public Health Agency of Sweden describes itself as “*an expert authority with responsibility for public health agencies at a national level. The Agency develops and supports activities to promote health, prevent illness and improve preparedness for health threats. Our vision statement: a public health that strengthens the positive development of society.”* It has a national responsibility for public health issues. One of the tasks of the agency is to ensure that the population is protected against communicable diseases and other threats including antibiotic resistance, addictions (including alcohol, smoking, gambling) and mental health (suicide prevention).

According to the Regulations of the Parliament (*Myndighetens instruction*) (1 §), the Swedish Public Health Agency shall work for good public health; evaluate the effects of methods and strategies in the field of public health; monitor the health situation in the population and factors that affect this through knowledge building and dissemination of knowledge; promote health; prevent diseases and injuries; and work for effective infection control and prevent disease dissemination (*smittspridning*). The work must be based on science.(2019c)

The Public Health Agency was formed on January 1, 2014 through merging the Swedish Institute for Communicable Disease Control (Smittskyddsinstitutet) and the Swedish National Institute of Public Health. In 2009, Johan Carlson was appointed as Director-General of the Swedish Institute for Communicable Disease Control and played a pivotal role in this merge. He moved on to the same function at the Public Health Agency in 2014 and onwards, and he was also a management board member of the European Centre for Disease Control (ECDC) during the pandemic.

The merge heavily reduced the academic research focus of the Swedish Institute for Communicable Disease Control. Six scientifically active professors at Karolinska Institutet with positions as senior consultants at the Public Health Agency were dismissed as consultants in 2014 and had to move their research labs to Karolinska Institutet (parasitologist Mats Wahlgren, virologist Jan Albert, vaccine researcher Peter Liljeström, clinical bacteriologist Lars Engstrand, epidemiologist Johan Giesecke, and immunologist Markus Maeurer).(Carlquist, 2020) On July 1, 2015, the Public Health Agency also assumed the overall responsibility for the country’s communicable disease control from the National Board of Health and Welfare (*Socialstyrelsen*). The Public Health Agency is also described as “the national focal point concerning international threats to public health”. The prioritised target groups for the agency are the Parliament and the Government; Governmental Agencies; Regions; Municipalities; County Administrative Boards; and “various organizations”.

In the US and most European countries they still have a separate Institute for Communicable Disease Control, with a sole focus on infection control – in contrast to the current Swedish Public Health agency, which is much broader and involves political and ideological concerns (including 'equality'), resulting in no one advocating specifically and exclusively for disease control.

The Public Health Agency has approximately 600 employees yet it seems they lack satisfying competence in social and behavioural sciences including psychology and sociology.(Sörbring, 2021) To support the Director-General, there is a “transparency council” (*insynsråd*) with a maximum of 10 members appointed by the Government, and the Director-General as chair. This council “exercises transparency in its operations and provides good advice” and has no decision-making powers.

The agency also has a State Epidemiologist (*statsepidemiolog)*, a position created in 1955, and a deputy State Epidemiologist. The State Epidemiologist is supposed to coordinate overall monitoring and analyses of the development of communicable diseases; and infection control – nationally and internationally. The State Epidemiologist is also responsible for the analyses of the consequences of infection control for the individual, health care and society. In addition to the Director-General, the main spokesperson of the Public Health Agency during the pandemic was the State Epidemiologist, Anders Tegnell, appointed in 2013 by the Director-General; and protégé of Johan Giesecke. Giesecke was State Epidemiologist between 1995-2005, and still an advisor to the World Health Organization (WHO) and paid consultant for the Public Health Agency during the pandemic – and one of the most vocal defenders of the Swedish strategy nationally and internationally. The State Epidemiologist between 2005-2013 was Annika Linde.(Ronge, 2020)

*The National Board of Health and Welfare*

The National Board of Health and Welfare (Socialstyrelsen [SoS], including SMER Socialstyrelsen Medical Ethics Committee, works to ensure good health, social welfare and high-quality health and social care on equal terms for the whole Swedish population. The activities concern social services, health and medical care, and communicable disease prevention”).

**The Swedish Association of Local Authorities and Regions (SALAR)** (*Sveriges kommuner och regioner, SKR)* is an employers' organization that supports the development of the regions’ and municipalities’ operations. All of Sweden's regions and municipalities are members of SALAR, which is a politically run organization with Anders Knape as President. A formal complaint has been submitted to *Coronakommissionen* by *Läkarförbundet* (Swedish union for medical doctors), urging the Commission to look into the role SALAR has been given during the pandemic. According to *Läkarförbundet*, the Government has tried to influence the governing of the health care system through SALAR instead of through the official agencies and channels. Since SALAR isn’t an official agency it doesn’t fall under the Freedom of Information Laws and hence lack the transparency meant for these procedures.(Cerberg, 2021, 2021x)

## **Supplement 4: Global, European, and Swedish timelines**

*Early global timeline*

The Wuhan Municipal Health Commission in Wuhan City, Hubei province, China, reported a cluster of 27 pneumonia cases (including seven severe cases) of unknown aetiology on December 31, 2019 and Wuhan went into lockdown on January 27, 2020. On January 30, 2020 the WHO(2021ad) declared the COVID-19 outbreak a public health emergency of international concern (PHEIC), the WHO’s highest level of alarm.(2021ac, 2021ad) At that time 98 cases and no deaths were reported in 18 countries outside China; with evidence of human-to-human transmission in four countries (8 cases) outside China (Germany, Japan, US, and Vietnam).(2021ad) On March 11, the WHO declared that COVID-19 should be considered a pandemic – recommending “detect, test, treat, isolate, trace, and mobilize their people in the response”.(2021ad) At this time, there were more than 118,000 diagnosed cases in 114 countries, and 4,291 people had lost their lives.(2021ad). The WHO also issued a series of recommendations on March 16, 2020. These recommendations included advice to increase preparedness, and to implement stronger infection prevention measures. They also recommended the “test and trace” strategy (Note: co-author Johan Giesecke).(Bedford et al., 2020)

*Early European timeline*

Although there could have been an increased EU role in the preparedness, monitoring and coordination of health emergencies since the 2000s, the individual countries unilaterally adopted different strategies, including “a series of uncoordinated border closures, varying confinement and testing strategies and national measures restricting the free circulation of masks”.(Beaussier and Cabane, 2020) For over 20 years, there has been a network of national public health agencies, and an early warning and response system; and in 2004 the ECDC was created.(Beaussier and Cabane, 2020)

According to the ECDC(2021ac), the first cases reported in Europe were diagnosed on January 24, 2020 in France and January 27-28, 2020 in Germany, all related to visits to China. On February 7, the ECDC issued a statement to plan for sufficient personal protective equipment (PPE) and to ensure surge capacity procedures were in place; and on February 8, 2020 the ECDC issued guidelines for Non-pharmaceutical countermeasures (including respiratory and environmental hygiene, and actions requiring the engagement of communities and the involvement of local, regional, or national authorities, i.e., social distancing and travel-related measures.(2021ac) On February 22, the Italian authorities reported on clusters with apparent local transmission (also in hospitals), with several cases reported in other European countries during the week after – link to visits to Italy and cases without links to Italy, China, or other countries with ongoing transmission. On March 17, 2020, the ECDC recommended social distancing and to “stay at home”, to “flatten the curve”. On March 25, 2020 all EU/EEA countries were affected (and more than 150 countries worldwide).(2021ac) On April 1, 2020 the ECDC recommended timely and accurate testing; and on April 8^th^ facemask use in the public to prevent asymptomatic infection.(Vogel, 2020, 2021ac)

*Early Swedish timeline*

Early interaction with ECDC

On January 31, 2020, the Swedish Minister for Health and Social Affairs (Lena Hallengren) visited the ECDC, located in Stockholm to discuss the situation; the first European diplomatic/political visit to the ECDC related to this pandemic.(2021ac) On February 26, 2020 the Swedish Minister for EU Affairs (Hans Dahlgren) visited ECDC, followed by the Swedish Prime Minister (Stefan Löfven) accompanied by State Epidemiologist Anders Tegnell, on March 3, 2020.(2021ac)

First weeks

The first COVID-19 case for Sweden was reported on January 31, 2020, and the first death on March 11, 2020 (**Figure 1**). Sweden was classified by the WHO among the countries with local transmission on March 2, 2020.(2020d, 2020t) The first press conference by the Public Health Agency was on March 4, 2020; and they have issued regular (initially several times a week) press releases since then in 2020 and 2021 and continues now in 2022.(2020-2021c) There were also regular media appearances and press conferences by key persons of the Public Health Agency. On March 7, one of the largest socio-cultural events in Sweden, the Melody Festival (Swedish preselection for Eurovision) was still organised with an audience (3 shows 25-30,000 visitors to each show) in the then epi-centre of Sweden, Stockholm.(Myhren, 2020, Westling et al., 2020, Domellöf-Wik, 2020) A few days after (mid-March) public gatherings of more than 500 individuals were banned, and end of March the limit was 50 individuals (yet these rules did not apply for private events).(Orlowski and Goldsmith, 2020) Sweden has reacted relatively late and less-stringently than most European countries.(Narlikar and Sottilotta, 2021, Cross et al., 2020)

Implemented measures

Swedish authorities never enforced a quarantine for those traveling from high-risk areas; nor was family quarantine initially recommended for household members of those who tested positive for COVID-19 or those with high risk contacts.(2020, 2020h, Vogel, 2020) On October 1, 2020 the Public Health Authority announced new ''rules of conduct'' which stated that an infection control physician could recommended to an individual that household members stay home if another was positive. However, this was not a uniform rule and State Epidemiologist Anders Tegnell specifically said that this was not for the individual to decide but rather that physicians could advise it in an individual assessment.(2020, 2020h) Even if the special rule of conduct was advised by a physician, people infected with COVID-19 and their household members could continue to bring and collect their children at day care/preschool and school, go to the grocery store and pharmacy, according to Anders Tegnell and the Public Health Agency – since this was never considered as quarantine or isolation as in other countries. Children in day care/pre-school and all students from primary school up to and including high school were also exempt from the conduct recommendation.(2020, 2020h) On June 16, 2020, the disease control officer for Stockholm stated that people with confirmed COVID-19 could still go to work if they had only mild symptoms.(Sörensen, 2020)

Restaurants, bars, gyms, and other businesses could stay open throughout the pandemic although opening hours and number of guests per table were restricted at some points. Although a lockdown was never in place, there was a reduction in mobility in Sweden – although less extreme than in other countries.(Cot et al., 2021, Sadowski et al., 2021, Sulyok and Walker, 2021, Toger et al., 2021, Vannoni et al., 2020) Many individuals and companies changed their social behaviour, suggesting an important voluntary social restraint.(Cot et al., 2021, Nordberg and Mattsson, 2020) Several companies in Sweden (e.g. Spotify) introduced distance working early in 2020 which was eventually also recommended by the Public Health Agency - although first criticized and discouraged, and relatively late compared to other countries (**Figure 1**).(Hiselius and Arnfalk, 2021, Nordberg and Mattsson, 2020)

There was also an expansion of the rights and flexibility to be in sick leave, including the cancellation of the *karensdag* (i.e., no salary on first day of illness).(2020q)

Infection control laws during the pandemic

COVID-19 was recognized as a disease dangerous to society and public health on February 1, 2020 (active from February 2), providing opportunities to take “extraordinary” measures such as quarantine and isolation of infectious or sick people at risk of spreading the coronavirus.(2020l, 2020n, Nordberg and Mattsson, 2020) The Public Health Agency changed their injunctions about the reporting of COVID-19-cases several times during 2020, basically limiting the detection and reporting of cases. According to The Communicable Diseases Act (S*mittskyddslagen*) a physician is obliged to report a notifiable disease promptly, even if just suspected. From May 5, 2020, ONLY physicians working at a laboratory or who were performing autopsies were to report cases (HSLF-FS 2020:23),(2020g) even though the regional infection control physicians could decide differently for their respective region, To what extent this was done is not known. Otherwise, to limit who can report a disease and to exclude suspected cases, reduces the number of cases that can be reported to the authorities and makes it more difficult to keep track of infections. From October 1, 2020, *suspected* cases of COVID-19 should NOT be reported at all (HSLF-FS 2020:45).(2020g)

Amendments to the Communicable Diseases Act (SFS 2020:241) was valid from April 18, 2020 until July 1, 2020, but was never imposed and the Government let it expire without asking for an extension.(2020b) When the infection rate increased, the Government did not have the legal means to impose strict measures, and it was not until January 10, 2021, that a new, temporary law (SLF: 2021:4) valid until the end of September, 2021, was passed.(2021u) At the time of writing, this has still not been implemented other than restricting opening hours and number of guests at restaurants and events, and without any legal repercussions.

The Royal Family

The Royal Family has no legislative or operational power in Sweden, but they were visible during the pandemic and had a few public confrontations with the Public Health Agency. In early March 2020, a school in Stockholm was temporarily closed after a confirmed COVID-19 case – the school attended by the daughter of the Crown Princess. (Sörensen, 2020) Anders Tegnell was publicly worried about this, since other schools may follow the example and he was clear about his desire to prevent any school closures.(Sörensen, 2020) The Queen and King also asked during a visit to the Karolinska Hospital on October 13, 2020 why facemasks were not recommended in Sweden – after which the physicians and Tegnell argued they were not effective and can even increase the risk.(Bjorklund, 2020b) The King spoke out on December 17, 2020 on national television, saying that he felt the Swedish strategy had failed: “We have a large number who have died and that is terrible.”(2020a) The Royal Family initiated a charitable memorial service on the pandemic’s anniversary evening (March 11, 2021) from the palace chapel, transmitted live by the national broadcasting company.(2021s, Bergmann, 2021)

General Advice (Allmänna Råd)

April 1, 2020, the Swedish Public Health Agency issued consolidated official “advice” to the public and businesses and organisations (HSLF-FS 2020:12). This was titled *The Public Health Agency’s regulations and public advice regarding everyone’s responsibility to prevent the infection of Covid-19 etc*. While theoretically obligatory, there is no monitoring or enforcement mechanisms for this advice.

These recommendations consolidated earlier advice and were regularly reissued with minor modifications but remained in place throughout 2020.

***All organisations in Sweden***

Based on recommendations from The Public Health Agency and regional Infection Control Doctors, all organisations in Sweden should take appropriate measures to prevent the spread of COVID-19. Measures may include the following –

1. providing information to members, staff, customers and other visitors
2. marking distances on the floor,
3. rearranging or otherwise creating space to avoid crowding,
4. holding digital meetings,
5. providing the opportunity to wash hands with soap and water and offers hand sanitizer; and
6. avoiding multiple people gathering, especially in crowded rooms

***Every individual in Sweden***Everyone has a responsibility to prevent the spread of COVID-19. To limit the spread, everyone should -

1. take care of their hand hygiene and wash their hands frequently with soap and water for at least 20 seconds,
2. keep their distance from each other indoors and outdoors in places where people congregate, such as shops, shopping centres, museums, libraries, service offices, and waiting rooms,
3. keep their distance from each other on public transport and other means of public transport,
4. avoid attending major social events such as parties, funerals, christenings, parties and weddings
5. keeping a distance from each other in sports grounds, baths, gyms and other exercise facilities and avoid changing in public changing rooms
6. avoid travelling during rush hours; and
7. refrain from unnecessary travel

***People over 70 and other risk groups***

To avoid the spread of COVID-19, persons over 70 years of age and those belonging to other risk groups should, in addition to what is mentioned above should -

1. limit their social contacts,
2. avoid travelling by public transport and other means of public transport; and
3. avoid shopping in shops such as pharmacies and grocery stores or spending time in other places where people gather.

***Persons infected or suspected of being infected by COVID-19***

To protect others from infection, anyone who knows they are infected or who has symptoms of COVID-19 should –

1. stay at home; and
2. avoid social contact.

***Workplaces***

To avoid the spread of COVID-19, employers should ensure that staff, if possible -

1. keep their distance from each other,
2. are able to wash their hands regularly with soap and water or otherwise use hand sanitizer,
3. work from home,
4. avoiding unnecessary travel for work; and
5. are able to adjust their working hours to avoid travelling during peak periods

**The Swedish Prison and Probation Service, the Swedish Migration Board and the National Board of Institutional Care**

To avoid the spread of COVID-19, these institutions should -

1. have in its management system procedures for preventing the spread of infection,
2. carry out regular risk assessments based on recommendations from the Public Health Agency and infection control doctors; and
3. develop instructions for staff and residents on how to prevent the spread of infection.

**Public transport**

To avoid the spread of COVID-19 on public transport, companies should –

1. ensure that traffic can run at the level necessary to reduce the risk of congestion,
2. limit the number of passengers per vehicle; and
3. inform their passengers on how to reduce the risk of transmission.

**Retail Outlets**

To avoid the spread of COVID-19 in retail outlets, shopping centres, shops, department stores, and similar establishments -

1. limit the number of customers present on the premises at any one time; and
2. develop alternative solutions to checkout queues or indicate how far customers should stand between each other

**Organisations**

To avoid the spread of COVID-19, associations should -

1. if possible, postpone annual meetings and general assemblies or conduct them digitally; and
2. avoid other meetings or conduct them digitally.

**Sports clubs**

To avoid the spread of the virus, sports clubs should -

1. ensure that close contact between athletes is avoided,
2. whenever possible, hold training sessions and other sports activities outdoors,
3. postpone matches, training matches, competitions and cups,
4. limit the number of spectators or otherwise avoid crowding; and
5. ensure that unnecessary travel in connection with sporting activities is avoided.

## **Supplement 5: Testing and contact-tracing in Sweden**

Testing was another weak point of the Swedish strategy.(2021b, Pashakhanlou, 2021) Even while community spread was reported all over Europe, only symptomatic individuals coming from COVID-19 hotspots could receive testing.(2021ac) Wider spread testing for active infections was only available from June 2020 and onwards and for symptomatic individuals only. This was mainly due to the Public Health Agency not prioritizing testing as was also clear from an email conversation between the Public Health Agency and the Ministry of Health and Social Affairs on March 30, 2020 – claiming “very high costs” as an argument.(Bjorklund, 2020a) Other emails suggested that testing was no priority for the Public Health Agency and the Government before widespread testing was already implemented in neighbouring Denmark.(Bjorklund, 2020a) Many academic research groups and companies offered help to increase the testing capacity early during the pandemic – yet this offer for help was usually declined by the Public Health Agency. In mid-March, two professors at Karolinska Institutet (Lars Engstrand) and the Royal Institute of Technology (Mathias Uhlén) already coordinated the air-delivery of additional equipment and personnel from China to drastically increase the testing-capacity in Sweden – before any approvals or support of their universities or the Public Health Agency.(Törnwall, 2020) Although this effort turned into the national COVID-19 testing centre later on, also financially supported by the Public Health Agency, it was not fully operative for months because of testing policies, bureaucracy and recommendations.

May 8, 2020, Professor Harriet Wallberg from *Karolinska Institutet* was appointed as a national testing coordinator; yet less than a month later she resigned.(J/TT, 2020) Her task was to increase testing, since the Government’s target of 100,000 tests by Mid-May(Nyström and Mossige-Norheim, 2020) was far from reached. At the end of May, only 36,000 tests in total were carried out in Sweden.(J/TT, 2020)

Instead of increasing its testing ability, as proposed by ECDC and WHO, Sweden always kept strict testing criteria, and access to testing was often complicated with long waiting times. Restricting who could report new cases of COVID-19 and removing “suspected cases”, resulted in underreporting of the number of infections, and Sweden quickly lost control of the spread of the disease. When testing capacities finally did increase, self-testing (without professional assistance) was mostly used and recommended by the Public Health Agency based on a small pilot study with both self-testing and professional testing,(2020e, Nyström and Mossige-Norheim, 2020, 2020i) with the risk of getting poor quality samples and false negative results. To date, priority for sampling is still for diagnostic sampling in healthcare and care for suspected cases (so not for contacts or potential cases).(2020i)

***Contact-tracing*** was also limited, although mandatory by the Communicable Diseases Act – and the responsibility of the infection control physicians.(Fagerlund, 2020) Instead of educating contact tracers, the limited testing reduced the need for tracing. In most places contact tracing was left to the infected individual without follow-up by the regional infection control physicians.(Fagerlund, 2020) Even if they did call their contacts, quarantine was not recommended, not even for household contacts (until October 1, 2020, and only for adults)(2020h). Even after, quarantine or testing were not recommended or mandatory after high-risk contacts or for suspected cases, especially not for children or those with prior infection (in the last 6 months).

On July 23, 2020, the Public Health Agency revoked its injunction (HSLF-FS 2015:4) about contact tracing and it was replaced by “Guidance for contact tracing of covid” (*Vägledning för smittspårning av covid-19*),(2020-21) which has been changed many times after that.(Fagerlund, 2020) Up until July 23, 2020 contact tracing by, or at least overseen by, medical personnel was mandatory by law, but was not imposed. Yet, the Public Health Agency placed the responsibility for contact tracing on the patient ill with COVID-19, since according to Anders Tegnell, the State Epidemiologist, this self-tracing works really well in Sweden for the sexually transmitted disease *Chlamydia* (as stated in August 2020),(Fagerlund, 2020) yet contact tracing is not supposed to be done by the individual for *Chlamydia* either. The Public Health Agency does not have jurisdiction over the regional disease control officers (*Regionala Smittskydd)*, which are independent, and basically does not have the right to block regional infection control physicians to conduct contact tracing (something that has happened in Uppsala).(2021l)

By ignoring testing, reporting, contact tracing and quarantine, COVID-19 was left to spread unnoticed at first. To plan what to do and to control the spread became increasingly difficult, as the number of infected individuals and the death toll show.

## **Supplement 6: Consequences of the Swedish approach in health- and elderly care.**

*Healthcare*

As mentioned in **Supplement 1**, Sweden had among the lowest number of ICU beds/capita in Europe before the pandemic.(Bauer et al., 2020) Although the number of ICU beds was increased during spring 2020, bed availability remained limited in several regions and was not transparently communicated to the public. The media and National Board of Health and Welfare also reported on the high survival of hospitalised and ICU patients,(Funck, 2020, 2020p, Strålin et al., 2021) with insufficient critical assessment of the potential selection bias of patients due to restricted access and triage. A field hospital was erected in Stockholm (*Älvsjö Fältsjukhus*) by a company (with Johan Giesecke’s wife among the stakeholders) yet it was not used.(Putilov, 2020) There were staff shortages in health- and elderly care all over the country.(2021g, Rolander, 2020)

*Elderly care*

This practice of restricting access to potentially live saving treatment in elderly individuals implies that the lives of several could have been saved or prolonged if they would have received oxygen treatment (not made available in many elderly homes) instead of morphine.(Habib, 2020, Sörensen, 2020, Vogel, 2020, Bjorklund and Ewing, 2020) This practice was reported in at least 6 of the 21 Swedish regions as shown by an independent investigation (see below),(Bjorklund, 2020b, 2020r, 2020o) and seemed to have attracted more international than national attention in the media.(Savage, 2020, Vogel, 2020, Bjorklund and Ewing, 2020) Although morphine is considered a palliative treatment, this practice cannot be called palliative care either, defined by the WHO as “Relieving serious health-related suffering, be it physical, psychological, social, or spiritual” in case of life-threatening illness,(2021af) since COVID-19 does not result in 100% lethality for elderly if adequate healthcare is available. This cannot be described as euthanasia, which is illegal in Sweden, since this implies termination of life by a physician at the repeated request of a person.(Materstvedt and Kaasa, 2002) Euthanasia is strictly regulated in the few countries, such as the Netherlands, where it is allowed (e.g. requiring evaluation by at least two physicians to determine irreversible suffering and administration by a physician).(Materstvedt and Kaasa, 2002) Non-voluntary and involuntary “euthanasia” are illegal in all countries, meaning *unrequested* end-of-life treatment in cognitively incompetent and competent individuals respectively.(Materstvedt and Kaasa, 2002) These are also termed “Life-terminating Acts Without the Explicit Request of a patient (LAWER)”.(Materstvedt and Kaasa, 2002)

Involuntary medicalised life-termination involves cognitively competent persons, either without asking about his or her will regarding euthanasia, or by going against his/her will in this respect, explicit or presumed. This is “plain murder” under Dutch law, where “standard” euthanasia is allowed.(Materstvedt and Kaasa, 2002) Therefore, every COVID-19 related case of “life-ending without explicit request” in Sweden would be expected to be assessed thoroughly, with potential prosecution for suspected murder or at least manslaughter, since it is a violation of the autonomy of the individual.(Materstvedt and Kaasa, 2002)

Both Johan Giesecke and Anders Tegnell, the former and current State Epidemiologists, have been advising many other countries advocating against lockdowns and restrictive measures, even after official reports clearly showed how this approach fails to protect the elderly and vulnerable.(McCurry, 2020, Giesecke, 2020, Vogel, 2020, Bjorklund and Ewing, 2020)

## **Supplement 7: Children and schools**

Even if the risk of getting severe illness is lower among children, some children do get severe illness and may die or develop long-COVID.(Bjurwald, 2021) A Professor in Paediatrics, Jonas Ludvigsson, is a researcher with close and regular (informal) contact with Anders Tegnell and the Public Health Agency.(Lindblad et al., 2021, 2020-2021b, Höög and Adman, 2020, Vogel, 2021, Ludvigsson, 2020a) He has been among the main supporters (or even initiators as one of the first signatories) of the Great Barrington Declaration (GBD) and the Swedish strategy.(Kulldorff et al., 2020) Ludvigsson has strongly opposed school closures as illustrated in a controversial correspondence published in the New England Journal of Medicine (February 21, 2021).(Besançon et al., 2021, Ludvigsson, 2021, Ludvigsson et al., 2021, Takefuji, 2021, Ludvigsson, 2020a, Vogel, 2021, Kulldorff et al., 2020)

Neither the mainstream media nor the Government have shown great interest in this important age-group and the potential short and long-term effects on their physical, mental and emotional health.(Bjurwald, 2021, Höög and Adman, 2020, 2021c) In addition, a large proportion of kindergarten teachers, primary school teachers (24%), secondary school teachers (15%) and school counsellors have been confirmed infected with COVID-19 (with 60% reporting to be infected at work); and several have died (yet official numbers are not available).(Hedman, 2021, Besançon et al., 2021, 2021p, TT, 2021) Primary school teachers were reported as the worst affected occupational group during autumn 2020,(TT, 2021) although the Public Health Agency still declared there was no increased risk among teachers in November 2020.(2020f, Hedman, 2021, Besançon et al., 2021, 2021p, TT, 2021)

## **Supplement 8: Scientific Advisory Groups during the pandemic**

*Karolinska Institutet’s expert group*

The group initially consisted of Jan Albert (infectious disease control, clinical virology, Professor), Anna Färnert (infectious disease specialist, molecular epidemiology, malaria, Professor), Johan Giesecke (infectious disease specialist, epidemiology, Emeritus Professor), Hedvig Glans (head of inpatient care Karolinska Hospital, PhD student), Sara Gredmark Russ (Infectious Disease specialist, Associate Professor), Ali Mirazimi (virology research, Adjunct Professor), Johan von Schreeb (Global Disaster Medicine, Professor) and Matti Sällberg (Dentist, Biomedical Analyst, Professor). The group’s tasks, among other things, were to provide support to the university management when it came to preventive measures and internal communication.(Ottersen, 2020)

From the beginning of the pandemic several of the members (Johan Giesecke, Johan von Schreeb, Jan Albert, Matti Sällberg and Ali Mirazimi) often appeared in the media as independent experts, promoting the Swedish strategy.(2020-2021d, Nilsson, 2020a, Nilsson, 2020b, Torkelsson, 2020, Sällström, 2020) E-mails (obtained through the Freedom of Information Laws) have shown that the group was in close contact with the Public Health Agency (located at the same premises in Stockholm), from the start and discussed the strategy, and how to respond to critique.(Karlsten, 2020b) This was not disclosed publicly.(Karlsten, 2020b) Neither was the fact that Giesecke, from March 30, 2020, was a paid consultant at the Public Health Agency, or that Ali Mirazimi is employed by the same agency.(2020j, Karlsten, 2021, Karlsten, 2020a) Giesecke, the former State Epidemiologist and member of the WHO’s STAG-group (Strategic And Technical Advisory Group) during the pandemic, still appeared as an independent expert on television, and claimed no conflicts of interests in a scientific publication published online on May 5, 2020, not mentioning his paid consultancy position.(Giesecke, 2020) This Lancet article was published on May 30 and heavily criticized for its claims that everyone will get infected, that lockdowns do not work, and that an unrealistically high immunity was already reached in Stockholm. [14,116-118,158,159] By then it had been made public that Giesecke was not independent, as he had claimed.(2020j) On May 27 his contract and invoices were obtained through the Freedom of Information Laws and published.(2020j) This showed that Johan Giesecke had even billed the Public Health Agency for the appearances he had made on television (as an *independent* expert).(2020j) When this was made public, the Public Health Agency announced that it was a “mistake”.(2020j, Karlsten, 2020a)

*The Royal Academy of Sciences*

The report from the Academy praised the Swedish healthcare system for success but pointed out that Sweden entered the pandemic with far fewer hospital beds and ICU beds per person than Europe or the neighbouring countries.(2020m) The report did not address clearly that the reason Sweden always had reserve beds in ICU was due to triage. Instead, it framed this as a serious ethical issue and stated, “The National Board of Health and Welfare has issued guidelines for these difficult assessments, "National principles for prioritizing routine medical care during the COVID-19 pandemic". It is unclear to what extent they needed to be used…”

The Academy report made several suggestions to protect the population when in public environments.(2020m) They recommended N95 masks be reserved for health care workers and more common surgical masks for use by the general population, mostly as source control. They also quoted WHO and recommended social distancing and hand washing. They noted, but did not challenge, that the Public Health Agency of Sweden did not recommend facemasks in public environments at that time. The report in August stated that test, trace, and isolation were powerful methods to prevent the spread of the COVID-19 and recommended this be carried out as well as banning travel.(2020m)

The Academy report briefly compared the mitigation strategy used in Sweden to the containment strategy applied in the neighbouring countries but did not take a stand on which was better at that time.(2020m)

The reports state, “We must therefore wait at least one year before we can give a definite answer to the question of whether Sweden's approach has been successful or has failed. Meanwhile, the waves of debate are likely to be high.”(2020m) The early working group’s report ended with a series of questions rather than strong recommendations.

The formation of an expert group and an advisory reference group was completed on September 24, 2020. This expert group led by Staffan Normark produced its first report on November 19, 2020 with work still in progress in 2021 with several updates.(2020s)

The November 19 report, “Measures to reduce the spread of COVID-19” differs from the recommendations of the Public Health Agency in several topics:(2021g)

- The expert group stated there was strong evidence for transmission of the virus by smaller droplets in the air (although they did not call them aerosols).
- They then agreed that masks should be recommended to limit infection, especially in public transport as well as health and elderly care.
- They also clearly stated that good ventilation with air exchange was important to limit spread.

*Science Forum Covid-19*

Between March 5 and April 13, 2020, at least 24 critical texts (debate articles and letters to the editor) were published in major Swedish newspapers in different constellations, however without having much of a visible impact.(2021ae) The scientists mainly criticized the lack of action and the lack of transparency regarding data and models. They argued for more precautionary measures such as quarantine for returning travellers, a comprehensive test-trace-isolate strategy, school closures and a short lockdown. Furthermore, they discussed the importance of transmission by infected people without symptoms and how people in risk groups needed to be better protected with adequate protection equipment, especially in elderly care.

Through their critical debate articles, the scientists became more and more aware of each other and 22 of them decided to write a debate article together, which was then published in Dagens Nyheter on April 14, 2020.(2020c) This article drew more attention for two main reasons: 1) the tone (“Officials, who so far have not shown any talent for either predicting or limiting the developments we now live with") and 2) the number of deaths mentioned.(2020c) On April 24, 2020, a clarification was added to this article to better explain the source for the data (ECDC) since the Public Health Agency disputed this official data. Several media outlets discussed the article and its content, and the criticism of the Public Health Agency was immediately dismissed by Anders Tegnell.(Connolly, 2020, Bjurwald et al., 2021, Vogel, 2020) Worth mentioning is the Corona Special from the state-sponsored Swedish Science Radio (*Vetenskapsradio*) which was broadcast on April 15, 2020.(Dingertz, 2020) The Review Board for Radio and Television later criticized this program for an unreasonable claim. The commentator said that the people behind the article subsequently corrected information on the number of COVID-19-related deaths in Sweden.(Dingertz, 2020) The Review Board is of the opinion that the Swedish Science Radio has not shown grounds for this.(Dingertz, 2020) The program therefore contravenes the requirement of objectivity.(Dingertz, 2020) Indeed, the official number of COVID-19 deaths later published by the National Board of Health and Welfare (104.33 per day on average) were very close to the number quoted by the 22 scientists (105 per day) while Tegnell’s estimate (about 60 per day) for the same period (April 7-9, 2020) was far off.(2020-2021a) (Connolly, 2020, Bjurwald et al., 2021, Vogel, 2020)

The group wrote many debate articles, recorded video recordings of online discussions, published scientific papers and gave interviews to the national and international media throughout the year of 2020 and beyond.(2021ae) In April 2021, two of the scientists obtained journalist passes to ask questions at press conferences of the Public Health Agency and government.

**Supplement 9: Systemic violation of the rule of law and human rights**

During the pandemic there have been major systemic violations of the rule of law and international human rights(1948)– evidence of a clear breakdown of the legal system.

Even outside of the scope of the pandemic, democracy, human rights and the judiciary’s independence do not receive strong protection in the Swedish constitution.(2021d)

The EU Charter of Fundamental Rights has direct legal implications for Swedish authorities, stating: Article 51 (Right to Health Care): “Everyone has the right of access to preventive health care and the right to benefit from medical treatment under the conditions established by national laws and practices. A high level of human health protection shall be ensured in the definition and implementation of all Union policies and activities.”

1. ***Limited and unequal access to prevention/protection***: Authorities have discouraged (or even prohibited) teachers, pupils, healthcare workers and other professionals from protecting themselves with facemasks or other measures. People who could not work from home and avoid public transport have been unable to protect themselves, in particular the socio-economically most vulnerable.(2021d) Individuals with disabilities, those in need of home-care or living in group or elderly homes were disproportionally affected during the pandemic as seen by their over-representation in the death statistics.(2021d) This violates the law as well as basic human rights; and specifically for children, the UN Convention on the Rights of the Child (*Barnkonventionen*). This is also in violation of the Communicable Diseases Act, which gives citizens the right to protect themselves against community spread of infections which are a danger to society.(2021l)
2. Continued ***lack of preparedness and planning***: Schools across the country, elderly homes and home care fail to have a Risk Assessment Plan (*Riskbedömningsplan*), also for the new COVID-19 strains, which violates the Work Environment Act (*Arbetsmiljölagen*) overseen by the Swedish Work Environment Authority (*Arbetsmiljöverket*). This is a systemic violation of the law, but there is little or no monitoring or enforcement and there are no repercussions.
3. ***Restricted and unequal access to healthcare***: Elderly have been denied healthcare in several regions and were effectively locked out from the basic institutions of the welfare state. This is a systematic discrimination and a violation of the human rights of the elderly. In at least six of the Swedish regions, elderly people were involuntarily euthanised with morphine and denied potentially life-saving oxygen without medical examination or any communication with the patient or his/her family. The Swedish Government is obliged to protect the human rights of all its citizens. This means that the Government is expected to take action to protect all lives (Article 2 “Right to life”, European Convention on Human Rights).(1948) There is strong scientific evidence, and therefore “a common consensus” in legal terms, to show that the current Swedish strategy (not including strong suppressive approaches to reduce the spread of the infection) is not the best to protect the lives of ALL its citizens. For all European citizens living in Sweden, healthcare is also a right. The passive Swedish strategy has endangered the access to a functioning healthcare system since no healthcare system has an endless capacity. The Swedish Government cannot claim it took sufficient and timely action to guarantee our access to healthcare - since sufficient scientific evidence has been available in time which was disregarded. Neighbouring countries have all taken stronger action weeks earlier than Sweden.
4. ***Infection control***:(2021l) The Regional Infection Control physicians are legally obliged to control the spread of infection, and e.g., implement a track-and-trace strategy, coordinated on a national level by the Public Health Agency – yet this never happened nationwide, and testing was restricted and with suboptimal procedures and access (violation of the Communicable Diseases Act). All individuals are also obliged to limit the spread of infection in society, but this has not been enforced.
5. ***Freedom of expression*** and lack of accurate information ***restricting public discourse***.(2021d) The European Convention of Human Rights - Article 10 - Freedom of expression protects both the freedom of expression AND the freedom of information, implies that everyone should be able to freely speak and express their opinions, and go to the public with these opinions, including criticizing government action. Even more, action against government rule is at the origin of this right and concerns the essence of the right. Both the European Convention on Human Rights and EU law protect in that respect going public with information that is of general concern, even against the regulations or orders of the employer (European Court of Human Rights case Guja v. Moldova, 2008, § 72), especially since there is an important overriding interest of public safety. Finally, there is the ***right of Academic Freedom***, “which should guarantee freedom of expression and action, freedom to communicate information”, as well as freedom to “seek and unrestrictedly disseminate knowledge and truth, which also came under strain during this pandemic.(Vrielink et al., 2010)

#

# **References**

1948. United Nations: Universal Declaration of Human Rights <https://www.un.org/en/about-us/universal-declaration-of-human-rights>; <https://www.echr.coe.int/Documents/Convention_ENG.pdf>

2005. Sveriges Riksdag (Swedish Parliament): Sverige och tsunamin - granskning och förslag Statens offentliga utredningar (Sweden and the tsunami - review and proposals The governments official investigations) 2005:104 <www.katastrofkommissionen.se>; <https://www.riksdagen.se/sv/dokument-lagar/dokument/statens-offentliga-utredningar/sverige-och-tsunamin---granskning-och-forslag_GTB3104d2>

2007. Regeringskansliet (Governments Offices): Krishantering i Regeringskansliet: Rapport från utredaren Christina Salomonson om inrättandet av en nationell krishanteringsfunktion i Regeringskansliet (Crisis management in the Government Offices: Report from investigator Christina Salomonson on the establishment of a national crisis management function in the Government Offices) <https://www.regeringen.se/49b6d5/contentassets/b9b5c9f1754b48448a73e7e00e443fe1/krishantering-i-regeringskansliet>

2015a. OECD Health Statistics: Country Note: How does health spending in Sweden compare?

2015b. *Offentlighetsprincipen (Governments Offices of Sweden: The principles of openness). Published Nov 4, 2014 (Updated Apr 1, 2015)*. Available from: <https://www.regeringen.se/sa-styrs-sverige/grundlagar-och-demokratiskt-deltagande/offentlighetsprincipen/>

2015c. *Regeringskansliet - Offentlighetsprincipen (Governments Offices of Sweden: The principles of openness). Published Nov 4, 2014 (Updated Apr 1, 2015)*. Available from: <https://www.regeringen.se/sa-styrs-sverige/grundlagar-och-demokratiskt-deltagande/offentlighetsprincipen/>

2018a. *The Global Goals and the 2030 Agenda for Sustainable Development. Published Jul 6, 2018*. Available from: <https://www.government.se/government-policy/the-global-goals-and-the-2030-Agenda-for-sustainable-development/> [Accessed 15 JUL 2021].

2018b. Hospitals in Europe Healthcare data. European Hospital and Healthcare Federation. .

2018c. Sweden to create new authority tasked with countering disinformation. *The Local Sweden*, Jan 15, 2018. Available from: <https://www.thelocal.se/20180115/sweden-to-create-new-authority-tasked-with-countering-disinformation/>

2019a. European Health Information Gateway (World Health Organisation): Bed occupancy rate (%), acute care hospitals only.

2019b. Eurostat - Statistics Explained - Healthcare resource statistics- beds.

2019c. Socialdepartementet (Ministry of Social Affairs): Sveriges Riksdag: Förordning (2013:1020) med instruktion för Folkhälsomyndigheten. Socialdepartementet (The Swedish Parliament: Ordinance (2013: 1020) with instructions for the Swedish Public Health Agency. Ministry of Social Affairs) <https://www.riksdagen.se/sv/dokument-lagar/dokument/svensk-forfattningssamling/forordning-20131020-med-instruktion-for_sfs-2013-1020>

2019d. *Statistics Sweden (SCB): Population statistics*. Available from: <http://www.statistikdatabasen.scb.se/pxweb/sv/ssd/> [Accessed 27 March 2020].

2020a. Coronavirus: Swedish King Carl XVI Gustaf says coronavirus approach 'has failed'". *BBC News*, Dec 17, 2020. Available from: <https://www.bbc.com/news/world-europe-55347021>

2020b. Department of Social Affairs: Svensk författningssamling: Lag om ändring i smittskyddslagen (2004:168) (Swedish Code of Statutes Act amending the Communicable Diseases Act (2004: 168))(Issued Apr 16, 2020; Published Apr 17, 2020) <https://svenskforfattningssamling.se/sites/default/files/sfs/2020-04/SFS2020-241.pdf>

2020c. DN Debatt. The 22 researchers: ”Folkhälsomyndigheten har misslyckats - nu måste politikerna gripa in” (The public health authority has failed - now politicians must intervene). *Dagens Nyheter*, April 14, 2020 (Updated April 24, 2020). Available from: <https://www.dn.se/debatt/folkhalsomyndigheten-har-misslyckats-nu-maste-politikerna-gripa-in>

2020d. Fjorton dagar som förändrade Sverige (Fourteen days that changed Sweden). *Ledarsidorna - alltid i opposition (The leadership webside - always in opposition)*.

2020e. Folkhälsomyndigheten (Public Health Agency): Egenprovtagning covid-19 för personal inom hälso- och sjukvård samt omsorg (Self-sampling covid-19 for staff in health and medical care and care)(20084-2) <https://www.folkhalsomyndigheten.se/contentassets/8aa82224a5c44ed3807bb49914f6ee87/egenprovtagning-covid-19-personal-halso-sjukvard-omsorg.pdf>

2020f. Folkhälsomyndigheten (Public Health Agency): Lärare har ingen högre risk för att smittas av covid-19 (Teachers have no higher risk of being infected with covid-19) <https://www.folkhalsomyndigheten.se/nyheter-och-press/nyhetsarkiv/2020/november/larare-har-ingen-hogre-risk-for-att-smittas-av-covid-19/>

2020g. Folkhälsomyndigheten (The Public Health Agency): Ändring i föreskrifter (HSLF-FS 2015:7) om anmälan av anmälningspliktig sjukdom i vissa fall (Amendment in regulations (HSLF-FS 2015: 7) on notification of notifiable illness in certain cases). (Decision Apr 28, 2020) <https://www.folkhalsomyndigheten.se/publicerat-material/publikationsarkiv/h/hslf-fs-202023/>

2020h. Folkhälsomyndigheten (The Public Health Agency): Personer som bor med smittade av covid-19 ska betraktas som möjliga fall. (People living with covid-19 infected should be considered as possible cases) Published Oct 1, 2020.

2020i. Folkhälsomyndigheten (The Public Health Agency): Utvärdering av egenprovtagning som metod för påvisning av SARS-COV-2 - Ett pilotprojekt (Evaluation of self-sampling as a method of detection of SARS-COV-2 - A pilot study) (01369-2020); Stöd inför etablering av rutiner för egenprovtagning för covid-19 (Support for the establishment of routines for self-sampling for covid-19) <https://www.folkhalsomyndigheten.se/smittskydd-beredskap/utbrott/aktuella-utbrott/covid-19/information-till-varden/personal-inom-halso--och-sjukvard/stod-infor-etablering-av-rutiner-for-egenprovtagning-for-covid-19/>

2020j. Giesecke har miljonavtal med Folkhälsomyndigheten – nu dras faktura tillbaka (Giesecke has a million agreement with the Swedish Public Health Agency - now the invoice is withdrawn). *Dagens Nyheter*, May 27, 2020. Available from: <https://www.dn.se/nyheter/sverige/giesecke-har-miljonavtal-med-folkhalsomyndigheten-nu-dras-faktura-tillbaka/>

2020k. Government Offices of Sweden - Ministry of Justice: Public access to information and secrecy - The legislation in brief <https://www.regeringen.se/4a76f3/contentassets/2c767a1ae4e8469fbfd0fc044998ab78/public-access-to-information-and-secrecy.pdf>

2020l. Krisinformation: Regeringen klassar coronavirus som samhällsfarlig sjukdom (Government classifies coronavirus as disease dangerous for the public) <https://www.krisinformation.se/nyheter/2020/februari/regeringen-klassar-corona-som-samhallsfarlig>

2020m. Kunliga Vetenskaps Akademien: Fakta och debatt om Covid-19. (The Royal Swedish Academy of Sciences: Facts and debate about Covid-19) May 15, 2020 (Updated Aug 28, 2020) <https://www.kva.se/sv/nyheter/fakta-och-debatt-om-covid-19>

2020n. Regeringens proposition 2019/20:144 Covid-19 och ändringar i smittskyddslagen (Covid-19 and amendments to the Communicable Diseases Act )(Apr 2, 2020)

2020o. Socialdepartementet, Regeringen: Regeringskanseliet: Granskning av äldreboenden presenterad. (Government Offices: Examination of nursing homes presented) Jul 7, 2020 <https://www.regeringen.se/artiklar/2020/07/granskning-av-aldreboenden-presenterad/?TSPD_101_R0=082953afa5ab20004ba88ffa4ab39ed8e5201bca4ded40bcf66de5ab4fc8797d623713cfa9a34325086554df241430005f7972f2e294ff885c146e4d669226d5f5e79e937e21b93bad917c74e957011ab2c3129313857e560b6795356dbfaac0>

2020p. Socialstyrelsen (National Board of Health and Welfare): Stor andel patienter har överlevt covid-19-vård på svenska sjukhus (A large proportion of patients have survived covid-19 care in Swedish hospitals). Jul 10, 2020 <https://www.socialstyrelsen.se/om-socialstyrelsen/pressrum/debattartiklar/stor-andel-patienter-har-overlevt-covid-19-vard-pa-svenska-sjukhus/>

2020q. Sweden: Government Temporarily Amends Sick Leave Rules During Coronavirus Outbreak. *Library of Congress*, Mar 20, 2020. Available from: <https://www.loc.gov/item/global-legal-monitor/2020-03-20/sweden-government-temporarily-amends-sick-leave-rules-during-coronavirus-outbreak/>

2020r. The Swedish Health and Care Inspectorate (IVO): Ingen region har tagit sitt fulla ansvar för individuell vård och behandling. (No region has taken full responsibility for individual care and treatment.) Nov 24, 2020 <https://www.ivo.se/publicerat-material/nyheter/2020/ingen-region-har-tagit-fullt-ansvar-for-individuell-vard/>

2020s. *Vetenskapsakademien bildar expertgrupp om Covid-19. (The Academy of Sciences forms an expert group on Covid-19) Sep 24, 2020*. Available from: <https://www.kva.se/sv/pressrum/pressmeddelanden/vetenskapsakademien-bildar-expertgrupp-om-covid-19>.

2020t. World Health Organistation: Coronavirus disease 2019 (COVID-19) Situation Report – 42 (Data as reported by 10AM CET 02 March 2020).

2020u. *World Values Survey*. Available from: <https://www.worldvaluessurvey.org/WVSOnline.jsp>.

2020-21. Folkälsomyndigheten (The Public Health Agency): Vägledning för smittspårning av covid-19 (Guidance for infection tracking of covid-19)(Published Jul 22, 2020 - last update Jun 16, 2021) <https://www.folkhalsomyndigheten.se/publicerat-material/publikationsarkiv/v/vagledning-for-smittsparning-av-covid-19/>

2020-2021a. Avlidna i covid-19 (Deaths from covid-19). April 27, 2020 - regularly updated since. .

2020-2021b. Folkhälsomyndigheten (The Public Health Agency): Covid-19 hos barn och unga – en kunskapssammanställning (Covid-19 in children and adolescents - a compilation of knowledge) (version 1-3) (Version 3 published Apr 15, 2021) <https://www.folkhalsomyndigheten.se/publicerat-material/publikationsarkiv/c/covid-19-hos-barn-och-unga--en-kunskapssammanstallning-version-3/>

2020-2021c. *Folkhälsomyndigheten: Nyhetsarkiv*. Available from: <https://www.folkhalsomyndigheten.se/nyheter-och-press/nyhetsarkiv/>.

2020-2021d. *KI (Karolinska Institutet) i medierna - arkiv (Karolinska Institutet in the media - archive)*. Available from: <https://nyheter.ki.se/ki-i-medierna-arkiv>.

2021a. The Care guide- Modern Health Care (Vårdguiden - Modern Hälso Vård) - all hospitals in Sweden <https://www.vardguiden.com/lista-over-sjukhus-i-sverige/>

2021b. CoronaKomissionen (Corona Comission) <https://coronakommissionen.com/>; <https://coronakommissionen.com/wp-content/uploads/2020/12/summary.pdf>

2021c. DN Debatt. ”Risk att en fjärde covidvåg i höst drabbar främst barn” ("Risk that a fourth covid wave this autumn mainly affects children"). *Dagens Nyheter*, Jun 2, 2021. Available from: <https://www.dn.se/debatt/risk-att-en-fjarde-covidvag-i-host-drabbar-framst-barn/>

2021d. EU Rule of Law Report Consultancy - Sweden Joint submission by the Swedish Section of the International Commission of Jurists and by Civil Rights Defenders <https://www.icj-sweden.org/eu-rule-of-law-report-consultancy-sweden-joint-submission-by-the-swedish-section-of-the-international-commission-of-jurists-and-by-civil-rights-defenders/>

2021e. *Governments Offices of Sweden: Sweden’s national life sciences strategy (published between Jan 21, 2019 - July 8, 2021 )*. Available from: <https://www.government.se/information-material/2020/11/swedens-national-life-sciences-strategy/> [Accessed July, 2021].

2021f. *Health and Social Care Inspectorate* Available from: <https://www.ivo.se/om-ivo/other-languages/english/>.

2021g. Kunliga Vetenskaps Akademien: Åtgärder för att minska spridningen av Covid-19. (Royal Swedish Academy of Sciences: Measures to reduce the spread of Covid-19) Feb 17, 2021.

2021h. *Media Landscapes: Expert Analyses of the State of Media: Sweden*. Available from: <https://medialandscapes.org/country/sweden> [Accessed Dec 2021].

2021i. Myndigheten för samhällsskydd och beredskap (MSB) (Swedish Civil Contingencies Agency): Dan Eliasson begär att få lämna uppdraget som generaldirektör för MSB. (Dan Eliasson requests to leave the position as Director General of MSB) Jan 6, 2021 <https://www.msb.se/sv/aktuellt/nyheter/2021/januari/dan-eliasson-begar-att-fa-lamna-uppdraget-som-generaldirektor-for-msb/>

2021j. *Myndigheten för samhällsskydd och beredskap, MSB - Psykologiskt försvar (The Swedish Civil Contingencies Agency, MSB - Psychological Defense)*. Available from: <https://www.msb.se/psykologisktforsvar> [Accessed Dec 2021].

2021k. Ny rapport: Privata försäkringspatienter går före i kön. (New report: Private insurance patients take the lead in the queue) Apr 4, 2020 (updated Aug 18, 2020 ). Dagens Nyheter.

2021l. *Peet Tüll - smittskyddslagen och smittskyddsläkarna (Peet Tüll - the Infection Control Act and the infection control doctors): Infektionsläkare och tidigare chef för Socialstyrelsens smittskyddsenhet svarar på frågor från er. (Infection doctor and former head of the National Board of Health and Welfare's infection control unit answers questions from you).* Available from: <https://www.facebook.com/VetenskapsforumCovid19/videos/peet-t%C3%BCll-smittskyddslagen-och-smittskyddsl%C3%A4karna/706694013532787/?__so__=permalink&__rv__=related_videos>.

2021m. *Public Health Agency of Sweden (Folkhälsomyndigheten)*. Available from: <www.folkhalsomyndigheten.se>.

2021n. Regeringen (Government); Riksdag (Parliament): Kommittédirektiv: Inrättande av Myndigheten för psykologiskt försvar. Beslut vid regeringssammanträde den 18 mars 2021 (Committee Directive: Establishment of the Swedish Defense Defense Agency. Resolution at the government meeting on March 18, 2021)(Dir. 2021:20) <https://www.regeringen.se/494e3d/contentassets/86b0598f69ae46fc9f2c4607a3f83cb2/inrattande-av-myndigheten-for-psykologiskt-forsvar-dir.-202120>; <https://www.riksdagen.se/sv/dokument-lagar/dokument/kommittedirektiv/inrattande-av-myndigheten-for-psykologiskt-forsvar_H9B120>; <www.sou.gov.se/wp-content/uploads/2020/05/SOU-2020_29_webb.pdf>; <https://www.regeringen.se/494e3d/contentassets/86b0598f69ae46fc9f2c4607a3f83cb2/inrattande-av-myndigheten-for-psykologiskt-forsvar-dir.-202120>

2021o. Regeringskanseliet (Governments Offices) <www.government.se>; <www.regeringen.se>

2021p. Smittan vanlig bland förskolepersonal – ”läget är enormt pressat” (Infection common among preschool staff - "the situation is under enormous pressure"). *Dagens Nyheter*, Jan 19, 2021. Available from: <https://www.dn.se/sverige/smittan-vanlig-bland-forskolepersonal-laget-ar-enormt-pressat/>

2021q. *Socialstyrelsen (The National Board of Health and Welfare)*. Available from: <https://www.socialstyrelsen.se/en>.

2021r. *Statistics Sweden*. Available from: <https://www.scb.se/en/>.

2021s. Sveriges Kungahus (Sweden's Royal House): Pandemins offer hedrade i Drottningholms slottskyrka (The victims of the pandemic were honored in Drottningholm's castle church).

2021t. *Sveriges Riksdag (Parliament)*. Available from: <www.riksdagen.se/en>.

2021u. Sveriges Riksdag (The Swedish Parliament): Lag (2021:4) om särskilda begränsningar för att förhindra spridning av sjukdomen covid-19 (Act (2021: 4) on special restrictions to prevent the spread of the disease covid-19). Issued Jan 8, 2021

2021v. Sveriges Riksdag (The Swedish Parliament): Smittskyddsförordning (Infection Control Ordinance) (2004:255; t.o.m. SFS 2019:1049) <https://www.riksdagen.se/sv/dokument-lagar/dokument/svensk-forfattningssamling/smittskyddsforordning-2004255_sfs-2004-255>

2021w. Sveriges Riksdag (The Swedish Parliament): Smittskyddslag (Infection Control Act) (2004:168; t.o.m. SFS 2020:430) <https://www.riksdagen.se/sv/dokument-lagar/dokument/svensk-forfattningssamling/smittskyddslag-2004168_sfs-2004-168>

2021x. *Swedish Association of Local Authorities and Regions*. Available from: <https://skr.se/skr/tjanster/englishpages.411.html>

2021y. *The Swedish Institute*. Available from: <https://si.se/en/>.

2021z. Swedish Institute: Healthcare in Sweden Swedish healthcare is largely tax-funded. And the overall quality is high. (Last update Jun 1, 2021) <https://sweden.se/life/society/healthcare-in-sweden>

2021aa. *The Swedish National Agency for Education*. Available from: <https://www.skolverket.se/>; <https://www.skolverket.se/andra-sprak-other-languages/english-engelska>.

2021ab. *Swedish Work Environment Authority*. Available from: <https://www.av.se/en/about-us/>.

2021ac. *Timeline of ECDC's response to COVID-19*. Available from: <https://www.ecdc.europa.eu/en/covid-19/timeline-ecdc-response> [Accessed 13 JUL 2021].

2021ad. *Timeline: WHO's COVID-19 response*. Available from: <https://www.who.int/emergencies/diseases/novel-coronavirus-2019/interactive-timeline> [Accessed 13 JUL 2021].

2021ae. *Vetcov-19 - Artiklar (Archive)*. Available from: <https://vetcov19.se/media/debattartiklar> [Accessed Aug, 2021].

2021af. World Health Organisation: Palliative Care.

ALUTTIS, C., CHIOTAN, C., MICHELSEN, M., COSTONGS, C. & BRAND, H. 2013. *Review of Public Health Capacity in the EU. Published by the European Commission Directorate General for Health and Consumers*.

APPELGREN, E. 2021. Media Management During COVID-19: Behavior of Swedish Media Leaders in Times of Crisis. *Journalism Studies*.

BAUER, J., BRUGGMANN, D., KLINGELHOFER, D., MAIER, W., SCHWETTMANN, L., WEISS, D. J., et al. 2020. Access to intensive care in 14 European countries: a spatial analysis of intensive care need and capacity in the light of COVID-19. *Intensive Care Medicine* 46: 2026-2034.

BEAUSSIER, A. L. & CABANE, L. 2020. Trust, Coordination and Multi-level Arrangements: Lessons for a European Health Union. *European Journal of Risk Regulation* 11: 808-820.

BEDFORD, J., ENRIA, D., GIESECKE, J., HEYMANN, D. L., IHEKWEAZU, C., KOBINGER, G., et al. 2020. COVID-19: towards controlling of a pandemic. *Lancet* 395: 1015-1018.

BERGMANN, S. 2020. Viruspolitik i en totalitär demokratur. (Virus politics in a totalitarian democracy). *Dagens Arena*, Dec 13, 2020. Available from: <https://www.dagensarena.se/essa/viruspolitik-en-totalitar-demokratur/>

BERGMANN, S. 2021. *Memoria Passionis Subversiva: The Moral Power of Remembrance in the Pandemic – in a Swedish lens,* Leipzig: EVA.

BESANÇON, L., STEADSON, D. & FLAHAULT, A. 2021. Open Schools, Covid-19, and Child and Teacher Morbidity in Sweden. *N Engl J Med* 384: e66.

BIDDLE, M. S. Y., GIBSON, A. & EVANS, D. 2021. Attitudes and approaches to patient and public involvement across Europe: A systematic review. *Health & Social Care in the Community* 29: 18-27.

BJORKLUND, K. 2020a. The Inside Story of How Sweden Botched Its Coronavirus Response. *Foreign Policy*, Dec 22, 2020. Available from: <https://foreignpolicy.com/2020/12/22/sweden-coronavirus-covid-response/>

BJORKLUND, K. 2020b. The Swedish Government’s Advice on Masks Is ‘’Fake News’’ in the Rest of the World. *Medium*, October 21, 2020. Available from: <https://kellybjorklund.medium.com/the-swedish-governments-advice-on-masks-is-fake-news-in-the-rest-of-the-world-4ab3d524eac4>

BJORKLUND, K. & EWING, A. 2020. The Swedish COVID-19 Response Is a Disaster. It Shouldn’t Be a Model for the Rest of the World. *Time*, Oct 14, 2020. Available from: <https://time.com/5899432/sweden-coronovirus-disaster/>

BJURWALD, L. 2021. Pandemins obekväma offer (The uncomfortable victims of the pandemic). *Dagens Arena*, July 17, 2021. Available from: <https://www.dagensarena.se/opinion/pandemins-obekvama-offer/>

BJURWALD, L., SCHÖNHOLZER, E. & ANDÉN, A. 2021. Näringslivets medieinstitut (business media institute): Maktens granskare eller maktens megafoner? Svensk journalistik under coronapandemin (Examiner of power or the megaphones of power? Swedish journalism during the corona pandemic) <https://naringslivets-medieinstitut.se/wp-content/uploads/2021/05/Coronarapporten-1.pdf>

BYLUND, P. L. & PACKARD, M. D. 2021. Separation of power and expertise: Evidence of the tyranny of experts in Sweden's COVID-19 responses. *Southern Economic Journal* 87: 1300-1319.

CARLQUIST, P. 2020. Coronapandemin: Elitforskarna som fick lämna FHM bryter tystnaden: Blev olyckligt (Corona pandemic: The elite researchers who were allowed to leave FHM break the silence: Became unhappy). *Aftonbladet* Jul 5, 2020 (updated Jul 6, 2020). Available from: <https://www.aftonbladet.se/nyheter/a/3Jredq/elitforskarna-som-fick-lamna-fhm-bryter-tystnaden-blev-olyckligt>

CERBERG, J. 2021. Läkarförbundet vill att SKR:s roll granskas av Coronakommissionen (The Swedish Medical Association wants SKR's role to be examined by the Corona Commission)(Published Aug 17, 2021 - updated Aug 25. 2021). *Läkartidningen*. Available from: <https://lakartidningen.se/aktuellt/nyheter/2021/08/lakarforbundet-vill-att-skrs-roll-granskas-av-coronakommissionen/>

CONNOLLY, P. 2020. Sweden’s top public health official slaps down researcher criticism. *Research Professional News*, Apr 20, 2020. Available from: <https://www.researchprofessionalnews.com/rr-news-europe-nordics-2020-4-sweden-s-top-public-health-official-slaps-down-researcher-criticism/>

COT, C., CACCIAPAGLIA, G. & SANNINO, F. 2021. Mining Google and Apple mobility data: temporal anatomy for COVID-19 social distancing. *Sci Rep* 11: 4150.

CROSS, M., NG, S. K. & SCUFFHAM, P. 2020. Trading Health for Wealth: The Effect of COVID-19 Response Stringency. *International Journal of Environmental Research and Public Health* 17.

DIDERICHSEN, F. 2021. How did Sweden Fail the Pandemic? *Int J Health Serv*: 20731421994848.

DINGERTZ, A. 2020. Myndigheten för press, radio och tv: SR fälls för osakligt uttalande om covid-19-debatt. (The authority for press, radio and television: SR is convicted for unreasonable statement about covid-19 debate.). *TT*, Nov 30, 2020. Available from: <https://via.tt.se/pressmeddelande/sr-falls-for-osakligt-uttalande-om-covid-19-debatt?publisherId=3235838&releaseId=3288084>

DOMELLÖF-WIK, M. 2020. Svenska Mellobeslutet: "Vi kör med publik" (Swedish Interim Decision: "We drive with an audience"). *Göteborgs Posten*, Mar 6, 2020. Available from: <https://www.gp.se/kultur/kultur/svenska-mellobeslutet-vi-k%C3%B6r-med-publik-1.24987198>

ESAIASSON, P., SOHLBERG, J., GHERSETTI, M. & JOHANSSON, B. 2020. How the coronavirus crisis affects citizen trust in institutions and in unknown others: Evidence from 'the Swedish experiment'. *European Journal of Political Research*.

ETZIONI, A. 2021. Community and COVID-19: Japan, Sweden and Uruguay. *Survival* 63: 53-76.

FAGERLUND, K. 2020. Covid-sjuka får smittspåra själva – nu höjs kritiska röster (Covid-ill should do own contact-tracing - criticicsm increases). *SVT Nyheter*, Aug 30, 2020. Available from: <https://www.svt.se/nyheter/inrikes/covidsmittade-far-smittspara-sjalva-nu-hojs-kritiska-roster>

FARINA, M. & LAVAZZA, A. 2020. Lessons From Italy's and Sweden's Policies in Fighting COVID-19: The Contribution of Biomedical and Social Competences. *Front Public Health* 8: 563397.

FUNCK, M. A. 2020. Åtta av tio coronapatienter överlever intensivvård (Eight out of ten corona patients survive intensive care). *Sveriges Radio*, Apr 12, 2020. Available from: <https://sverigesradio.se/artikel/7450992>

GERLE, E. 2021. Being, belonging, and borders: Scandinavian creation theology as political theology. *Dialog-a Journal of Theology* 60: 45-53.

GIESECKE, J. 2020. The invisible pandemic. *Lancet* 395: e98.

GRANBERG, M., RONNBLOM, M., PADDEN, M., TANGNAS, J. & OJEHAG, A. 2021. Debate: Covid-19 and Sweden's exceptionalism-a spotlight on the cracks in the social fabric of a mature welfare state. *Public Money & Management*.

GREVE, B., BLOMQUIST, P., HVINDEN, B. & VAN GERVEN, M. 2020. Nordic welfare states-still standing or changed by the COVID-19 crisis? *Soc Policy Adm*.

HABIB, H. 2020. Has Sweden's controversial covid-19 strategy been successful? *Bmj* 369: m2376.

HAYRY, M. 2021. The COVID-19 Pandemic: Healthcare Crisis Leadership as Ethics Communication. *Cambridge Quarterly of Healthcare Ethics* 30: 42-50.

HEDMAN, E. 2021. Ny studie: Var fjärde grund­skol­lärare smittad av covid-19: "Misstag att inte ta lärarnas oro på allvar" (New study: Every fourth primary school teacher is infected with covid-19. "Mistake not to take teachers' concerns seriously"). *Skolvärlden (School World)*, Apr 29, 2021. Available from: <https://skolvarlden.se/artiklar/ny-studie-var-fjarde-grundskollarare-smittad-av-covid-19>

HENLEY, J. 2021. Stefan Löfven back as Swedish PM weeks after no-confidence vote. *The Guardian*. Available from: <https://www.theguardian.com/world/2021/jul/07/stefan-lofven-back-as-swedish-pm-weeks-after-no-confidence-vote>

HISELIUS, L. W. & ARNFALK, P. 2021. When the impossible becomes possible: COVID-19's impact on work and travel patterns in Swedish public agencies. *European Transport Research Review* 13.

HÖÖG, J. & ADMAN, P. 2020. FHM:s rapport håller inte för vetenskaplig granskning (FHM's report is not suitable for scientific review). *Expressen*, Dec 2, 2020. Available from: <https://www.expressen.se/debatt/fhms-rapport-haller-inte-for-vetenskaplig-granskning/>

HUSA, J. 2011. The stories we tell ourselves about Nordic law in specific. *Isaidat Law Review* 1.

INGLEHART, R. 2018. Cultural Evolution: People’s motivations are changing, and reshaping the world. . Cambridge: Cambridge University Press.

J/TT, E. 2020. Sweden's coordinator for increasing Covid-19 testing finishes in role. *Sveriges Radio (Radio Sweden)*, Jun 3, 2020. Available from: <https://sverigesradio.se/artikel/7487696>

JOSEFSSON, K. W. 2021. Perspectives of Life in Sweden During the COVID-19 Pandemic. *Journal of Clinical Sport Psychology* 15: 80-86.

KARLSTEN, E. 2020a. Efter granskningen: DN och SVT korrigerar artiklar med Johan Giesecke (After the review: DN and SVT correct articles with Johan Giesecke). Available from: <https://emanuelkarlsten.se/efter-granskningen-dn-och-svt-korrigerar-artiklar-med-johan-giesecke/> [Accessed May 28, 2020].

KARLSTEN, E. 2020b. Tegnell-mejlen: Berättelsen om Johan Giesecke och Folkhälsomyndigheten (The Tegnell email: The story of Johan Giesecke and the Swedish Public Health Agency). Available from: <https://emanuelkarlsten.se/tegnell-mejlen-berattelsen-om-johan-giesecke-och-folkhalsomyndigheten/> [Accessed Aug 11, 2020].

KARLSTEN, E. April 8, 2021 2021. Vaccinexperten i medier också anställd på Folkhälsomyndigheten: ”Kände inte till det” (The vaccine expert in the media also employed at the Swedish Public Health Agency: "Did not know about it"). Available from: <https://emanuelkarlsten.se/vaccinexperten-i-medier-ocksa-anstalld-pa-folkhalsomyndigheten-kande-inte-till-det/>.

KORHONEN, J. & GRANBERG, B. 2020. Sweden Backcasting, Now?-Strategic Planning for Covid-19 Mitigation in a Liberal Democracy. *Sustainability* 12.

KULLDORFF, M., GUPTA, S. & BHATTACHARYA, C. 2020. *Great Barrington Declaration (Oct 4, 2020)*. Available from: <https://gbdeclaration.org/> [Accessed Aug 2021].

LANDO, O. 2001. Nordic Countries, a Legal Family? A Diagnosis and a Prognosis. *Global Jurist Advances* 1.

LARSSON, S.-O. & HARTIG, M. 2015. Ålderism (Ageism). *In:* (UPS), U. P. S. (ed.). Sweden.

LINDBLAD, S., LINDQVIST, A., RUNESDOTTER, C. & WÄRVIK, G. B. 2021. In education we trust: on handling the COVID-19 Pandemic in the Swedish welfare state. *Z Erziehwiss*: 1-17.

LINDSTRÖM, M. 2020. The COVID-19 pandemic and the Swedish strategy: Epidemiology and postmodernism. *SSM Popul Health* 11: 100643.

LINDSTRÖM, M. 2021. The New Totalitarians: The Swedish COVID-19 strategy and the implications of consensus culture and media policy for public health. *SSM Popul Health* 14: 100788.

LINELL, A., RICHARDSON, M. X. & WAMALA, S. 2013. The Swedish national public health policy report 2010. *Scand J Public Health* 41: 3-56.

LUDVIGSSON, J. F. 2020a. Children are unlikely to be the main drivers of the COVID-19 pandemic - A systematic review. *Acta Paediatr* 109: 1525-1530.

LUDVIGSSON, J. F. 2020b. The first eight months of Sweden's COVID-19 strategy and the key actions and actors that were involved. *Acta Paediatr*.

LUDVIGSSON, J. F. 2021. Open Schools, Covid-19, and Child and Teacher Morbidity in Sweden. Reply. *N Engl J Med* 384: e66.

LUDVIGSSON, J. F., ENGERSTRÖM, L., NORDENHÄLL, C. & LARSSON, E. 2021. Open Schools, Covid-19, and Child and Teacher Morbidity in Sweden. *N Engl J Med* 384: 669-671.

MATERSTVEDT, L. J. & KAASA, S. 2002. Euthanasia and physician-assisted suicide in Scandinavia--with a conceptual suggestion regarding international research in relation to the phenomena. *Palliat Med* 16: 17-32.

MCCURRY, C. 2020. Expert says Republic of Ireland should allow spread of Covid-19 among people under 60. *The Irish News*, Sep 23, 2020. Available from: <https://www.irishnews.com/news/republicofirelandnews/2020/09/23/news/expert-says-ireland-should-allow-spread-of-covid-19-among-people-under-60-2076059/>

MENS, H., KOCH, A., CHAINE, M. & BENGAARD ANDERSEN, A. 2021. The Hammer vs Mitigation-A comparative retrospective register study of the Swedish and Danish national responses to the COVID-19 pandemic in 2020. *Apmis*.

MYHREN, L. 2020. Corona stoppar inte svenska Melodifestivalen (Corona does not stop the Swedish Melodifestivalen). *Sveriges Radio*, Mar 6, 2020. Available from: <https://sverigesradio.se/artikel/7423218>

NANDA, M., AASHIMA & SHARMA, R. 2021. COVID-19: A Comprehensive Review of Epidemiology and Public Health System Response in Nordic Region. *International Journal of Health Services* 51: 287-299.

NARLIKAR, A. & SOTTILOTTA, C. E. 2021. Pandemic narratives and policy responses: west European governments and COVID-19. *Journal of European Public Policy*.

NIELSEN, J. H. & LINDVALL, J. 2021. Trust in government in Sweden and Denmark during the COVID-19 epidemic. *West European Politics*.

NILSSON, J. 2020a. Professor (Jan Albert): Rätt att inte stänga skolorna (Correct not to close schools) *Dagens Medicin*, March 13, 2020. Available from: <https://www.dagensmedicin.se/specialistomraden/infektion/professor-ratt-att-inte-stanga-skolorna/>

NILSSON, M. 2020b. CoronavirusetSvenska fallen: Albert: Spårning effektiv vid begränsad smitta (CoronavirusSwedish cases: Albert: Tracking effective in case of limited infection). *Omni*, May 28, 2020. Available from: <https://omni.se/albert-sparning-effektiv-vid-begransad-smitta/a/Qo1AGx>

NORDBERG, A. & MATTSSON, T. 2020. CoViD-19 pandemic in Sweden: measures, policy approach and legal and ethical debates. *Biolaw Journal-Rivista Di Biodiritto*: 731-739.

NYGREN, K. G. & OLOFSSON, A. 2021. Swedish exceptionalism, herd immunity and the welfare state: A media analysis of struggles over the nature and legitimacy of the COVID-19 pandemic strategy in Sweden. *Current Sociology*.

NYSTRÖM, M. & MOSSIGE-NORHEIM, T. 2020. Sveriges nya coronastrategi: ”Fler ska testas” (Sweden's new corona strategy: "More to be tested"). *Expressen*, Apr 30, 2020. Available from: Sveriges nya coronastrategi: ”Fler ska testas”

ORLOWSKI, E. J. W. & GOLDSMITH, D. J. A. 2020. Four months into the COVID-19 pandemic, Sweden's prized herd immunity is nowhere in sight: latest global population viral seropositivity findings have serious implications for the Scandinavian countries and beyond. *Journal of the Royal Society of Medicine* 113: 292-298.

OTTERSEN, O. 27 Feb, 2020 2020. Coronaviruset: KI etablerar covid-19-grupp (The coronavirus: KI establishes covid-19 group). Available from: <https://blog.ki.se/rektor/2020/02/27/coronaviruset-ki-etablerar-covid-19-grupp/?_ga=2.90578906.1790751704.1627045593-840892602.1627045593>.

PÅLSSON, A.-M. 2011. *Knapptryckarkompaniet : rapport från Sveriges riksdag*

PASHAKHANLOU, A. H. 2021. Sweden's coronavirus strategy: The Public Health Agency and the sites of controversy. *World Med Health Policy*.

PETRIDOU, E. & ZAHARIADIS, N. 2020. Staying at home or going out? Leadership response to the COVID-19 crisis in Greece and Sweden. *Journal of Contingencies and Crisis Management*.

PIERRE, J. 2020. Nudges against pandemics: Sweden's COVID-19 containment strategy in perspective. *Policy and Society* 39: 478-493.

PUTILOV, E. 2020. Gieseckes företag hade ledande roll i uppbyggnaden av fältsjukhuset i Älvsjö (Giesecke's company played a leading role in the construction of the field hospital in Älvsjö). *Samnytt*, June 1, 2020. Available from: <https://samnytt.se/gieseckes-foretag-hade-ledande-roll-i-uppbyggnaden-av-faltsjukhuset-i-alvsjo/>

RHODES, A., FERDINANDE, P., FLAATTEN, H., GUIDET, B., METNITZ, P. G., MORENO, R. P., et al. 2012. The variability of critical care bed numbers in Europe. *Intensive Care Med* 38: 1647-53.

ROLANDER, N. 2020. Sweden’s Covid Workers Are Quitting in Dangerous Numbers. *Bloomberg*, Dec 12, 2020. Available from: <https://www.bloomberg.com/news/articles/2020-12-12/swedish-covid-workers-are-quitting-leaving-icus-short-staffed>

RONGE, J. 2020. Folkhälsomyndigheten kan betala Giesecke en miljon. (The public health authority can pay Giesecke one million). *Expressen*, May 26, 2020. Available from: <https://www.expressen.se/nyheter/folkhalsomyndigheten-kan-betala-giesecke-en-miljon/>

ROSENBERG, G. 2002. Chapter 10: The Crisis of Consensus in Postwar Sweden. *Culture and crisis: the case of Germany and Sweden.* New York: Berghahn Books.

ROTHSTEIN, B. 2005 (online 2009). *Social Traps and the Problem of Trust*, Cambridge University Press.

SADOWSKI, A., GALAR, Z., WALASEK, R., ZIMON, G. & ENGELSETH, P. 2021. Big data insight on global mobility during the Covid-19 pandemic lockdown. *J Big Data* 8: 78.

SÄLLSTRÖM, H. 2020. Coronaviruset - Smittan i Norden Professor (Jan Albert): Lär bli jämna dödstal i Nordens länder (Coronavirus - Infection in the Nordic countries Professor (Jan Albert): Death rates in the Nordic countries will probably be similar). *Omni*, May 10, 2020. Available from: <https://omni.se/professor-lar-bli-jamna-dodstal-i-nordens-lander/a/xP2dJ8>

SAVAGE, M. 2020. Coronavirus: What's going wrong in Sweden's care homes? *BBC News*, May 19, 2020. Available from: <https://www.bbc.com/news/world-europe-52704836>

SOLANDER, I. 2020. Det får du göra och inte göra i familjeisolering. (You can do that and not do it in family isolation) *Dagens Nyheter*, Oct 1 2020 (Updated 14 Oct 14, 2020). Available from: <https://www.dn.se/sverige/det-far-du-gora-och-inte-gora-i-familjeisolering/>

SÖRBRING, K. 2021. Unika listan: Så rankas svenska coronaexperter (Unique list: How Swedish corona experts are ranked). *Expressen*, 4 May, 2021. Available from: <https://www.expressen.se/nyheter/unika-listan-sa-rankas-svenska-coronaexperter/>

SÖRENSEN, J. 2020. Terror in Utopia: crisis (mis-) management during the covid-19 pandemic in Sweden. *Sociološki pregled*: 961-1007.

STRÅLIN, K., WAHLSTRÖM, E., WALTHER, S., BENNET-BARK, A. M., HEURGREN, M., LINDÉN, T., et al. 2021. Mortality trends among hospitalised COVID-19 patients in Sweden: A nationwide observational cohort study. *Lancet Reg Health Eur* 4: 100054.

SULYOK, M. & WALKER, M. D. 2021. Mobility and COVID-19 mortality across Scandinavia: A modeling study. *Travel Med Infect Dis* 41: 102039.

TAKEFUJI, Y. 2021. Open Schools, Covid-19, and Child and Teacher Morbidity in Sweden. *N Engl J Med* 384: e66.

THEBAULT, R. 2021. Sweden prime minister’s abrupt resignation upends the country and its politics. *Washington Post*, Aug 23, 2021. Available from: <https://www.washingtonpost.com/world/2021/08/23/sweden-prime-minister-resigns/>

TOGER, M., KOURTIT, K., NIJKAMP, P. & OSTH, J. 2021. Mobility during the COVID-19 Pandemic: A Data-Driven Time-Geographic Analysis of Health-Induced Mobility Changes. *Sustainability* 13.

TORKELSSON, A.-C. 2020. Coronaviruset Svenska fallen: Experten (Jan Albert): 40 procent kan ha haft smittan i Stockholm (The coronavirus Swedish cases: The expert (Jan Albert): 40 percent may have had the infection in Stockholm). *Omni*, April 29, 2020. Available from: <https://omni.se/experten-40-procent-kan-ha-haft-smittan-i-stockholm/a/g7nyo0>

TÖRNWALL, M. 2020. Wallenbergs kupp – spelet bakom det unika coronalabbet (Wallenberg's coup - the game behind the unique coronalaboratory) *Nyheter Sverige*, May 23, 2020. Available from: <https://ki.se/media/108884/download>

TRÄGÅRDH, L. 2013. Chapter 10: The Historical Incubators of Trust in Sweden: From the Rule of Blood to the Rule of Law. *In:* REUTER, M., WIJKSTRÖM, F. & UGGLA, B. (eds.) *Trust and Organizations - Confidence across Borders.* New York: Nature America Inc. .

TT. 2021. Nya siffror: Grundskollärare värst drabbade av smitta (New figures: Primary school teachers worst affected by infection). *Läraren/TT*, Jan 19, 2021. Available from: <https://www.lararen.se/nyheter/coronaviruset/larare-betydligt-mer-smittade-an-ovriga-yrken>

VANNONI, M., MCKEE, M., SEMENZA, J. C., BONELL, C. & STUCKLER, D. 2020. Using volunteered geographic information to assess mobility in the early phases of the COVID-19 pandemic: a cross-city time series analysis of 41 cities in 22 countries from March 2nd to 26th 2020. *Global Health* 16: 85.

VOGEL, G. 2020. ‘It’s been so, so surreal.’ Critics of Sweden’s lax pandemic policies face fierce backlash. *Science Magazine*, Oct 6, 2020. Available from: <https://www.sciencemag.org/news/2020/10/it-s-been-so-so-surreal-critics-sweden-s-lax-pandemic-policies-face-fierce-backlash>

VOGEL, G. 2021. COVID-19 Data in paper about Swedish schoolchildren come under fire. *Science* 371: 973-974.

VRIELINK, J., LEMMENS, P. & PARMENTIER, S. 2010. Academic freedom as a fundamental right (The Leage of European Research universities -LERU- working group on human rights) <https://www.law.kuleuven.be/linc/onderzoek/ap6-academicfreedom-final-dec2010.pdf>

WENANDER, H. 2021. Sweden: Non-binding Rules against the Pandemic - Formalism, Pragmatism and Some Legal Realism. *Eur J Risk Regul* 12: 127-142.

WESTLING, F., THORNEUS, E. & EK, T. 2020. SVT i krismöte efter danska beslutet om Mello. SVT: ”Kommer köra i dagsläget” (SVT in crisis meeting after the Danish decision on Mello SVT: "Will drive in the current situation"). *Aftonbladet*, Mar 6, 2020. Available from: <https://www.aftonbladet.se/nojesbladet/melodifestivalen/a/8mkGld/svt-i-krismote-efter-danska-beslutet-om-mello>

WOELFERT, F. S. & KUNST, J. R. 2020. How Political and Social Trust Can Impact Social Distancing Practices During COVID-19 in Unexpected Ways. *Front Psychol* 11: 572966.
